# The Functional Redundancy of Neddylation E2s and E3s in Modulating the Fitness of Regulatory T Cells

**DOI:** 10.34133/research.0212

**Published:** 2023-08-18

**Authors:** Di Wu, Yi Sun

**Affiliations:** ^1^ Cancer Institute (Key Laboratory of Cancer Prevention and Intervention, China National Ministry of Education) of the Second Affiliated Hospital and Institute of Translational Medicine, Zhejiang University School of Medicine, Hangzhou 310029, China.; ^2^ Cancer Center of Zhejiang University, Hangzhou 310029, China.; ^3^ Zhejiang Provincial Clinical Research Center for Cancer, Zhejiang Province, China.; ^4^ Key Laboratory of Molecular Biology in Medical Sciences, Zhejiang Province, China.; ^5^ Research Center for Life Science and Human Health, Binjiang Institute of Zhejiang University, Hangzhou 310053, China.

## Abstract

Neddylation is necessary for activation of Cullin-RING ligases (CRLs), which degrade various immune regulatory proteins. Our recent study showed that while depletion of neddylation E2–E3 pair Ube2f-Sag in regulatory T (T_reg_) cells had no obvious phenotype, the same depletion of either Ube2m or Rbx1 caused inflammation disorders with different severity. Whether these E2s or E3s compensate each other in functional regulations of T_reg_ cells is, however, previously unknown. In this report, we generated *Foxp3*^*Cre*^;*Ube2m*^*fl*/*fl*^;*Ube2f*^*fl*/*fl*^ or *Foxp3*^*Cre*^;*Rbx1*^*fl*/*fl*^;*Sag*^*fl*/*fl*^ double-null mice by simultaneous deletion of both neddylation E2s or E3s in T_reg_ cells, respectively. Remarkably, *Ube2m&Ube2f* double-null mice developed much severe autoimmune phenotypes than did *Ube2m*-null mice, indicating that *Ube2m* markedly compensates *Ube2f* in T_reg_ cells. The minor worsened autoimmune phenotypes seen at the very early stage in *Rbx1&Sag* double-null than *Rbx1*-null mice is likely due to already severe phenotypes of the later, indicating a minor compensation of *Rbx1* for *Sag*. The RNA profiling-based analyses revealed that up- and down-regulations of few signaling pathways in T_reg_ cells are associated with the severity of autoimmune phenotypes. Finally, severer inflammation phenotypes seen in mice with double E3-null than with double E2-null T_reg_ cells indicate a neddylation-independent mechanism of 2 E3s, also known to serve as the RING component of CRLs in regulation of T_reg_ cell fitness.

## Introduction

Neddylation, a ubiquitination-like process, is catalyzed by an enzyme cascade, consisting of NEDD8 E1 activation enzyme, NEDD8 E2 conjugating enzyme, and NEDD8 E3 ligases. In mammalian cells, the heterodimer of NEDD8 activating enzyme E1 subunit 1 (NAE1/APPBP1) and ubiquitin like modifier activating enzyme 3 (UBA3/NAEβ) is the only one E1 found; ubiquitin conjugating enzyme E2 M (UBE2M, also known as UBC12) and ubiquitin conjugating enzyme E2 F (UBE2F) are 2 known E2s. Among over a dozen of E3s, Ring-box 1 (RBX1) and Ring-box 2 (RBX2, also known as SAG/RNF7/ROC2) are most well studied for cullin neddylation [1,2].

**Fig. 1.**
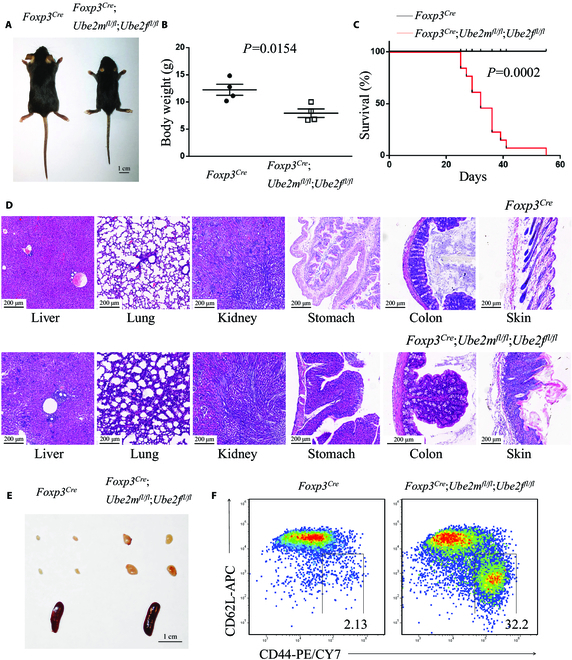
Deletion of *Ube2m* and *Ube2f* in T_reg_ cells leads to an early-onset fatal inflammatory disorder. (A) Representative images of *Foxp3*^*Cre*^ and *Foxp3*^*Cre*^;*Ube2m*^*fl*/*fl*^;*Ube2f*^*fl*/*fl*^ mice (p20, scale bar =1 cm). (B) Gross body weight of *Foxp3*^*Cre*^ and *Foxp3*^*Cre*^;*Ube2m*^*fl*/*fl*^;*Ube2f*^*fl*/*fl*^ mice (*n* = 4). (C) Survival curves of *Foxp3*^*Cre*^ and *Foxp3*^*Cre*^;*Ube2m*^*fl*/*fl*^;*Ube2f*^*fl*/*fl*^ mice (*n* = 13). (D) H&E staining of multiple organs from *Foxp3*^*Cre*^ and *Foxp3*^*Cre*^;*Ube2m*^*fl*/*fl*^;*Ube2f*^*fl*/*fl*^ mice (p19 to p20, scale bar = 200 μm). (E) Representative images of the peripheral lymph nodes (top) and spleen (bottom) from *Foxp3*^*Cre*^ and *Foxp3*^*Cre*^;*Ube2m*^*fl*/*fl*^;*Ube2f*^*fl*/*fl*^ mice (p22, scale bar = 1 cm). (F) Expression of CD44 and CD62L in T_con_ cells from peripheral lymph nodes of *Foxp3*^*Cre*^ and *Foxp3*^*Cre*^;*Ube2m*^*fl*/*fl*^;*Ube2f*^*fl*/*fl*^ mice (p21).

**Fig. 2.**
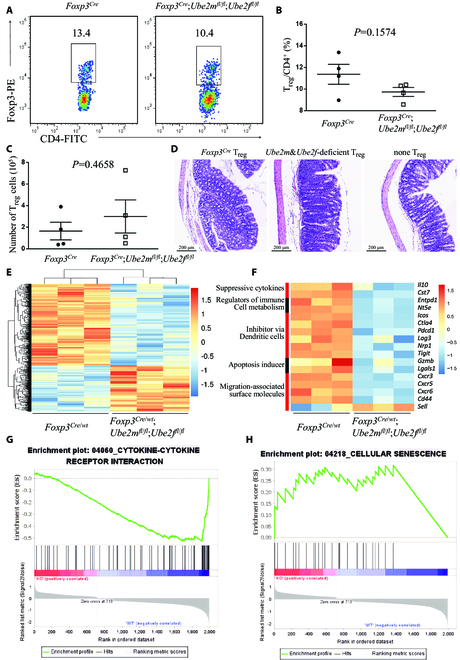
Impaired suppressive function of *Ube2m*&*Ube2f*-deficient T_reg_ cells. (A) Expression of Foxp3 in CD4^+^ T cells in peripheral lymph nodes from *Foxp3*^*Cre*^ and *Foxp3*^*Cre*^;*Ube2m*^*fl*/*fl*^;*Ube2f*^*fl*/*fl*^ mice at p20. (B) T_reg_/CD4^+^ ratios in peripheral lymph nodes from *Foxp3*^*Cre*^ and *Foxp3*^*Cre*^;*Ube2m*^*fl*/*fl*^;*Ube2f*^*fl*/*fl*^ mice (p19 to p21, *n* = 4). (C) T_reg_ cell numbers in peripheral lymph nodes from *Foxp3*^*Cre*^ and *Foxp3*^*Cre*^;*Ube2m*^*fl*/*fl*^;*Ube2f*^*fl*/*fl*^ mice (p19 to p21, *n* = 4). (D) Representative images of distal colon after H&E staining (scale bar = 200 μm). (E) Unsupervised cluster analysis of the transcriptional alterations in *Ube2m*&*Ube2f*-deficient T_reg_ cells compared to the *Foxp3*^*Cre*^ control T_reg_ cells with Fc > 1.5 and *P* < 0.05. (F) Differentially expressed genes related to T_reg_ cell function in CD4^+^YFP^+^ T_reg_ cells from *Foxp3*^*Cre*/*wt*^ and *Foxp3*^*Cre*/*wt*^;*Ube2m*^*fl*/*fl*^;*Ube2f*^*fl*/*fl*^ mice, determined by transcriptional profiling with Fc > 1.5 and *P* < 0.05. (G) Gene set enrichment analysis (GSEA) of cytokine–cytokine receptor interaction genes in CD4^+^YFP^+^ T_reg_ cells from *Foxp3*^*Cre*/*wt*^ and *Foxp3*^*Cre*/*wt*^;*Ube2m*^*fl*/*fl*^;*Ube2f*^*fl*/*fl*^ mice, determined by transcriptional profiling with Fc > 1.5 and *P* < 0.05. (H) GSEA of cellular senescence genes in CD4^+^YFP^+^ T_reg_ cells from *Foxp3*^*Cre*/*wt*^ and *Foxp3*^*Cre*/*wt*^;*Ube2m*^*fl*/*fl*^;*Ube2f*^*fl*/*fl*^ mice, determined by transcriptional profiling with Fc > 1.5 and *P* < 0.05.

**Fig. 3.**
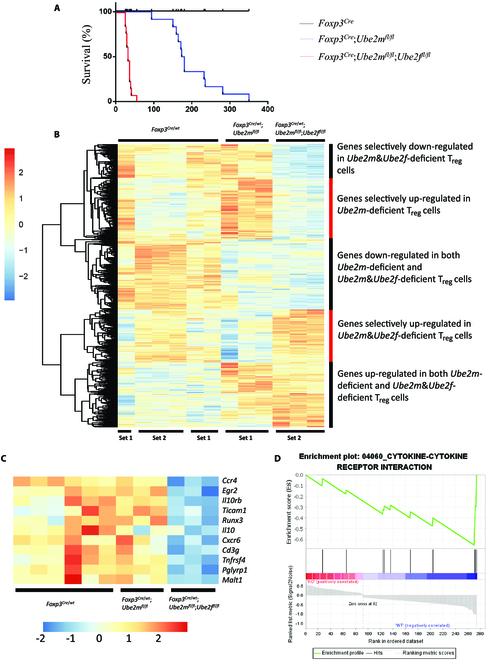
Enhanced inflammation phenotype and transcription alteration caused by *Ube2m*&*Ube2f* deficiency compared to *Ube2m* deficiency in T_reg_ cells. (A) Survival curves of *Foxp3*^*Cre*^, *Foxp3*^*Cre*^;*Ube2m*^*fl*/*fl*^ [15], and *Foxp3*^*Cre*^;*Ube2m*^*fl*/*fl*^;*Ube2f*^*fl*/*fl*^ mice. (B) Unsupervised cluster analysis of the transcriptional alterations in *Ube2m*&*Ube2f*- and *Ube2m*-deficient T_reg_ cells compared to the *Foxp3*^*Cre*^ control T_reg_ cells. (C) Genes related with T_reg_ cell function or regulation, and also down-regulated in *Ube2m*&*Ube2f*-deficient, but not *Ube2m*-deficient, T_reg_ cells. (D) GSEA of cytokine–cytokine receptor interaction genes altered in *Ube2m*&*Ube2f*-deficient, but not *Ube2m*-deficient, T_reg_ cells.

**Fig. 4.**
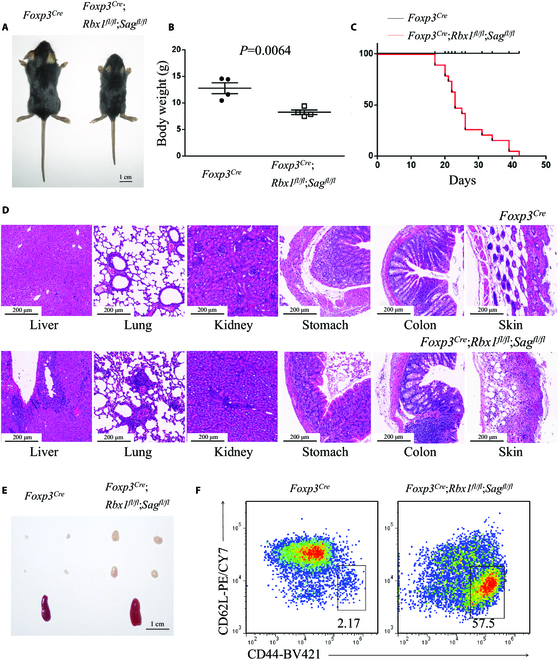
Deletion of *Rbx1* and *Sag* in T_reg_ cells leads to an early-onset fatal inflammatory disorder. (A) Representative images of *Foxp3*^*Cre*^ and *Foxp3*^*Cre*^;*Rbx1*^*fl*/*fl*^;*Sag*^*fl*/*fl*^ mice (p19, scale bar =1 cm). (B) Gross body weight of *Foxp3*^*Cre*^ and *Foxp3*^*Cre*^;*Rbx1*^*fl*/*fl*^;*Sag*^*fl*/*fl*^ mice (*n* = 4). (C) Survival curves of *Foxp3*^*Cre*^ and *Foxp3*^*Cre*^;*Rbx1*^*fl*/*fl*^;*Sag*^*fl*/*fl*^ mice (*n* = 19). (D) H&E staining of multiple organs from *Foxp3*^*Cre*^ and *Foxp3*^*Cre*^;*Rbx1*^*fl*/*fl*^;*Sag*^*fl*/*fl*^ mice (p20 to p21, scale bar = 200 μm). (E) Representative images of the peripheral lymph nodes (top) and spleen (bottom) from *Foxp3*^*Cre*^ and *Foxp3*^*Cre*^;*Rbx1*^*fl*/*fl*^;*Sag*^*fl*/*fl*^ mice (p19, scale bar = 1 cm). (F) Expression of CD44 and CD62L in T_con_ cells from peripheral lymph nodes of *Foxp3*^*Cre*^ and *Foxp3*^*Cre*^;*Rbx1*^*fl*/*fl*^;*Sag*^*fl*/*fl*^ mice (p20).

**Fig. 5.**
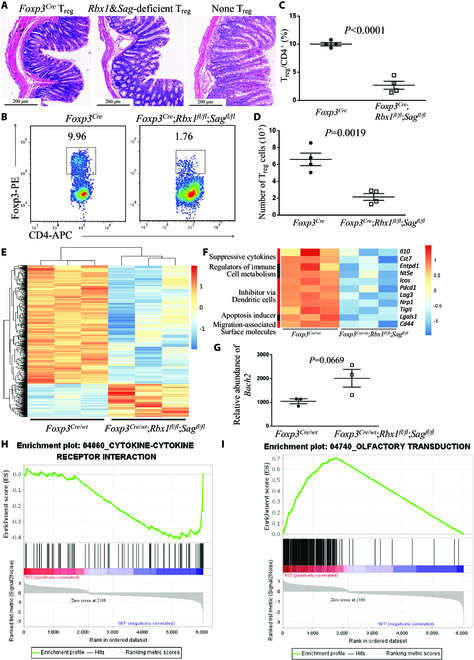
Impaired suppressive function of *Rbx1*&*Sag*-deficient T_reg_ cells. (A) Representative images of distal colon after H&E staining (scale bar = 200 μm). (B) Expression of Foxp3 in CD4^+^ T cells in peripheral lymph nodes from *Foxp3*^*Cre*^ and *Foxp3*^*Cre*^;*Rbx1*^*fl*/*fl*^;*Sag*^*fl*/*fl*^ mice at p20. (C) T_reg_/CD4^+^ ratios in peripheral lymph nodes from *Foxp3*^*Cre*^ and *Foxp3*^*Cre*^;*Rbx1*^*fl*/*fl*^;*Sag*^*fl*/*fl*^ mice (p19 to p20, *n* = 4). (D) T_reg_ cell numbers in peripheral lymph nodes from *Foxp3*^*Cre*^ and *Foxp3*^*Cre*^;*Rbx1*^*fl*/*fl*^;*Sag*^*fl*/*fl*^ mice (p19 to p20, *n* = 4). (E) Unsupervised cluster analysis of the transcriptional alterations in *Rbx1*&*Sag*-deficient T_reg_ cells compared to the *Foxp3*^*Cre*^ control T_reg_ cells with Fc > 1.5 and *P* < 0.05. (F) Differentially expressed genes related to T_reg_ cell function in CD4^+^YFP^+^ T_reg_ cells from *Foxp3*^*Cre*/*wt*^ and *Foxp3*^*Cre*/*wt*^;*Rbx1*^*fl*/*fl*^;*Sag*^*fl*/*fl*^ mice, determined by transcriptional profiling with Fc > 1.5 and *P* < 0.05. (G) Relative abundance of *Bach2* mRNA in CD4^+^YFP^+^ T_reg_ cells from *Foxp3*^*Cre*/*wt*^ and *Foxp3*^*Cre*/*wt*^;*Rbx1*^*fl*/*fl*^;*Sag*^*fl*/*fl*^ mice, determined by transcriptional profiling. (H) GSEA of cytokine–cytokine receptor interaction genes in CD4^+^YFP^+^ T_reg_ cells from *Foxp3*^*Cre*/*wt*^ and *Foxp3*^*Cre*/*wt*^;*Rbx1*^*fl*/*fl*^;*Sag*^*fl*/*fl*^ mice, determined by transcriptional profiling with Fc > 1.5 and *P* < 0.05. (I) GSEA of olfactory transduction genes in CD4^+^YFP^+^ T_reg_ cells from *Foxp3*^*Cre*/*wt*^ and *Foxp3*^*Cre*/*wt*^;*Rbx1*^*fl*/*fl*^;*Sag*^*fl*/*fl*^ mice, determined by transcriptional profiling with Fc > 1.5 and *P* < 0.05.

**Fig. 6.**
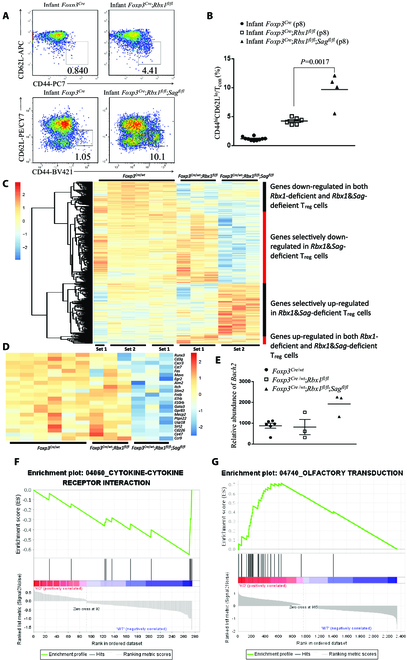
Enhanced inflammation phenotype and transcription alteration caused by *Rbx1*&*Sag* deficiency compared to *Rbx1* deficiency in T_reg_ cells. (A) Expression of CD44 and CD62L in T_con_ cells from peripheral lymph nodes of infant *Foxp3*^*Cre*^, *Foxp3*^*Cre*^;*Rbx1*^*fl*/*fl*^, and *Foxp3*^*Cre*^;*Rbx1*^*fl*/*fl*^;*Sag*^*fl*/*fl*^ mice (p8). The upper panel was cited from our previous study [15] for comparison purpose. (B) Proportion of CD44^hi^CD62^lo^ effector/memory cells among T_con_ cells in peripheral lymph nodes from infant *Foxp3*^*Cre*^, *Foxp3*^*Cre*^;*Rbx1*^*fl*/*fl*^, and *Foxp3*^*Cre*^;*Rbx1*^*fl*/*fl*^;*Sag*^*fl*/*fl*^ mice (p8). (C) Unsupervised cluster analysis of the transcriptional alterations in *Rbx1*&*Sag*- and *Rbx1*-deficient T_reg_ cells compared to the *Foxp3*^*Cre*^ control T_reg_ cells. (D) Genes related with T_reg_ cell function or regulation, and also down-regulated in *Rbx1*&*Sag*-deficient, but not *Rbx1*-deficient, T_reg_ cells. (E) Relative abundance of *Bach2* mRNA in CD4^+^YFP^+^ T_reg_ cells from *Foxp3*^*Cre*/*wt*^, *Foxp3*^*Cre*/*wt*^;*Rbx1*^*fl*/*fl*^, and *Foxp3*^*Cre*/*wt*^;*Rbx1*^*fl*/*fl*^;*Sag*^*fl*/*fl*^ mice, determined by transcriptional profiling. (F) GSEA of cytokine–cytokine receptor interaction genes altered in *Rbx1*&*Sag*-deficient, but not *Rbx1*-deficient, T_reg_ cells. (G) GSEA of olfactory transduction genes altered in *Rbx1*&*Sag*-deficient, but not *Rbx1*-deficient, T_reg_ cells.

**Fig. 7.**
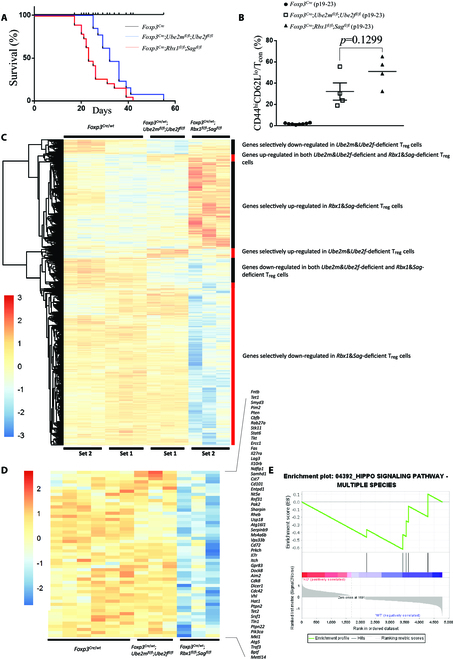
Enhanced inflammation phenotype and transcription alteration caused by *Rbx1*&*Sag* deficiency compared to *Ube2m*&*Ube2f* deficiency in T_reg_ cells. (A) Survival curves of *Foxp3*^*Cre*^, *Foxp3*^*Cre*^;*Ube2m*^*fl*/*fl*^;*Ube2f*^*fl*/*fl*^, and *Foxp3*^*Cre*^;*Rbx1*^*fl*/*fl*^;*Sag*^*fl*/*fl*^ mice; *P* = 0.0350 between the survivals of *Foxp3*^*Cre*^;*Ube2m*^*fl*/*fl*^;*Ube2f*^*fl*/*fl*^ mice and *Foxp3*^*Cre*^;*Rbx1*^*fl*/*fl*^;*Sag*^*fl*/*fl*^ mice. (B) Proportion of CD44^hi^CD62^lo^ effector/memory cells among T_con_ cells in peripheral lymph nodes from *Foxp3*^*Cre*^, *Foxp3*^*Cre*^;*Ube2m*^*fl*/*fl*^;*Ube2f*^*fl*/*fl*^, and *Foxp3*^*Cre*^;*Rbx1*^*fl*/*fl*^;*Sag*^*fl*/*fl*^ mice (p19 to p23). (C) Unsupervised cluster analysis of the transcriptional alterations in *Ube2m*&*Ube2f*- and *Rbx1*&*Sag*-deficient T_reg_ cells compared to the *Foxp3*^*Cre*^ control T_reg_ cells. (D) Genes related with T_reg_ cell function or regulation, and also down-regulated in *Rbx1*&*Sag*-deficient, but not in *Ube2m*&*Ube2f*-deficient, T_reg_ cells. (E) GSEA of Hippo signaling pathway altered in *Rbx1*&*Sag*-deficient, but not *Ube2m*&*Ube2f*-deficient, T_reg_ cells.

Cullin-RING ligases (CRLs) are the largest E3 ubiquitin ligase family, consisting of 4 subunits: (a) scaffold cullins, (b) adaptor proteins, (c) RING component (RBX1/2), and (d) substrate-recognizing receptor. There are 8 members of cullin family, including cullin-1 to cullin-3, cullin-4A and cullin-4B, cullin-5, cullin-7, and cullin-9, with cullin-1 to cullin-5 being most well studied, which are physiological substrates of neddylation. Cullin neddylation triggers the conformation change, leading to activation of CRLs [3]. The UBE2M-RBX1 E2–E3 pair catalyzes neddylation of cullin-1 to cullin-4, while the UBE2F-RBX2/SAG axis catalyzes cullin-5 neddylation [4]. In addition, RBX1 and RBX2/SAG also act as the RING component of cullin-1 to cullin-4 and cullin-5, respectively, for targeted ubiquitylation and subsequent proteasome degradation of numerous substrates [5–7]. Thus, RBX1 and RBX2/SAG are dual E3s for both neddylation and ubiquitylation mediated by CRLs.

Our previous studies revealed that during mouse embryonic development, Rbx1 and Sag/Rbx2 are functional nonredundant, since in mice total knockout of *Rbx1* or *Sag* leads to embryonic lethality at different stage with different mechanism [8,9]. Although whether Ube2m or Ube2f is also functionally redundant during development is currently unknown, the fact that neddylation of cullin-1 to cullin-4 versus cullin-5 by the Ube2m-Rbx1 pair versus the Ube2f-Sag pair [4] strongly suggests their functional independency. On the other hand, we recently showed a cross-talk between UBE2M and UBE2F, 2 neddylation E2s, in which UBE2M serves as a ubiquitylation E2 to promote ubiquitylation of UBE2F for proteasome degradation [10]. Whether there is a cross-talk between RBX1 and SAG remains unknown.

Regulatory T (T_reg_) cells are a suppressive subpopulation of CD4^+^ T lymphocytes, and transcription factor Foxp3 is the master marker of T_reg_ cells [11–13]. T_reg_ cells are essential for immune homeostasis, which is illustrated by the early-onset fatal inflammation caused by depletion of T_reg_ cells [14]. We recently investigated the role of neddylation-CRL system in T_reg_ cells using *Foxp3*^*Cre*^-*LoxP* system by generating 4 conditional knockout mouse models with deletion in T_reg_ cells of 2 neddylation E2s, *Ube2f* or *Ube2m*, or 2 dual E3s, *Sag* or *Rbx1*, individually [15]. Interestingly, mice with the deletion of *Ube2f* or *Sag* in T_reg_ cells had no visible phenotype, indicating that the Ube2f-Sag axis plays little, if any, role in regulation of T_reg_ cells under physiological condition. Meanwhile, mice with the deletion of *Ube2m* or *Rbx1* in T_reg_ cells suffered from severe autoimmune inflammatory phenotypes, indicating the functional requirement of the Ube2m-Rbx1 axis in T_reg_ cells [15]. The fact that much severer phenotypes are observed in Rbx1-null mice than in Ube2m-null mice indicates a neddylation-independent role of Rbx1 in T_reg_ cells. However, it is still unknown whether 2 neddylation E2s or E3s are redundant in functional regulation of T_reg_ cells.

In this study, we addressed this functionally redundant question by generating T_reg_ double knockout mouse models of 2 E2s, *Foxp3*^*Cre*^;*Ube2m*^*fl*/*fl*^;*Ube2f*^*fl*/*fl*^, or 2 E3s, *Foxp3*^*Cre*^;*Rbx1*^*fl*/*fl*^;*Sag*^*fl*/*fl*^, for simultaneous depletion of Ube2m and Ube2f or Rbx1 and Sag in T_reg_ cells, respectively. Both T_reg_ cell double knockout mice developed severe autoimmune disorders with an early-onset fatality. Given that Ube2f deletion in T_reg_ cells had no phenotype, much severe autoimmune phenotypes in *Ube2m&Ube2f* double deletion than in *Ube2m* single deletion in T_reg_ cells indicate that Ube2m compensates the function of Ube2f in T_reg_ cells. On the other hand, minor increased severity in T_reg_ *Rbx1&Sag* double deletion than *Rbx1* single deletion suggests a major role of Rbx1 in regulation of T_reg_ cell function. Furthermore, a greater severity in autoimmune phenotypes of mice with *Rbx1*&*Sag* deficiency than *Ube2m*&*Ube2f* deficiency in T_reg_ cells suggests a neddylation-independent function of Rbx1/Sag. The comparison of RNA profiling in T_reg_ cells with paired genotypes revealed a positive correlation of up- or down-regulation of few key signaling pathways with the severity of autoimmune phenotypes of T_reg_ cell knockout mice.

## Results

### Fatal inflammation in *Foxp3*^Cre^*Ube2m*^fl/fl^;*Ube2f*^fl/fl^ mice

We have previously reported the phenotypical changes caused by individual deletion of *Ube2m* or *Ube2f* in T_reg_ cells, and found that while the *Foxp3*^*Cre*^;*Ube2f*^*fl*/*fl*^ mice had no visible phenotypic changes, the *Foxp3*^*Cre*^;*Ube2m*^*fl*/*fl*^ mice developed severe inflammation disorders, where ~50% of mice died at about 4 months [15]. To study possible functional redundancy of 2 neddylation E2s in T_reg_ cells, we intercrossed the single-null mice [15] and *Foxp3*^*YFP*-*Cre*^ (*Foxp3*^*Cre*^) mice [16] to generate conditional knockout mice with deletion of both *Ube2m* and *Ube2f* in T_reg_ cells simultaneously (designated as “*Foxp3*^*Cre*^;*Ube2m*^*fl*/*fl*^;*Ube2f*^*fl*/*fl*^”). Strikingly, the *Foxp3*^*Cre*^;*Ube2m*^*fl*/*fl*^;*Ube2f*^*fl*/*fl*^ mice suffered from an early-onset alterations of appearance, including reduced body size, collapsed ears, festered skin (Fig. 1, A and B), and a significantly shortened life span with 100% of death rate at p55 (Fig. 1C). In *Foxp3*^*Cre*^;*Ube2m*^*fl*/*fl*^;*Ube2f* ^*fl*/*fl*^ mice at ~p20, the organ hematoxylin and eosin (H&E) staining revealed lymphocyte infiltration in liver, lung, kidney, stomach, colon, and skin (Fig. 1D). The autopsy revealed swollen peripheral immune organs, including lymph nodes and spleens, although the cellularity did not reach the statistically significant level (Fig. 1E and Fig. S1A). A more detailed characterization revealed a robust activation of immune cells from *Foxp3*^*Cre*^;*Ube2m*^*fl*/*fl*^;*Ube2f*^*fl*/*fl*^ mice (~p20), as evidenced by a decreased ratio of CD4^+^/CD8^+^ T cells (Fig. S1B) and increased proportion of effector/memory T cells (CD44^hi^CD62L^lo^, T_eff/mem_ cells) among conventional T cells (CD4^+^Foxp3^−^, T_con_ cells) (Fig. 1F and Fig. S1C). Such fatal inflammatory disorders recaptured the phenotypes observed in mice with depleted T_reg_ cells [14] in severity, indicating a pivotal role of neddylation E2s in the T_reg_ cells.

### Impaired suppressive functions and altered transcriptome of *Ube2m*&*Ube2f*-deficient T_reg_ cells

Given that both the T_reg_/CD4^+^ ratios (Fig. 2, A and B) and absolute numbers of T_reg_ cells (Fig. 2C) are largely similar in peripheral lymph nodes derived from either *Foxp3*^*Cre*^;*Ube2m*^*fl*/*fl*^;*Ube2f*^*fl*/*fl*^ or *Foxp3*^*Cre*^ control mice around p20, we hypothesized that the severe inflammation disorders observed in *Foxp3*^*Cre*^;*Ube2m*^*fl*/*fl*^;*Ube2f*^*fl*/*fl*^ mice at the same age is most likely attributable to the loss of the suppressive function of T_reg_ cells. To test this hypothesis, we performed an in vivo suppression assay in immunodeficient *Rag1*^−/−^ mice. Transfer of naive T (T_nai_) cells (CD4^+^Foxp3^−^CD44^lo^CD62L^hi^) into *Rag1*^−/−^ recipients led to a wasting disease with colitis within 4 weeks. We found that such colitis was prevented by simultaneous transfer of T_reg_ cells from wild-type mice, but not from *Ube2m*&*Ube2f*-deficient mice (Fig. 2D), demonstrating that double deletion of neddylation E2s impairs the suppressive function of T_reg_ cells.

To define the underlying mechanism, we performed RNA profiling and analyzed the transcriptome alterations caused by *Ube2m*&*Ube2f* deletion in T_reg_ cells. Considering that the inflammation in *Foxp3*^*Cre*^;*Ube2m*^*fl*/*fl*^;*Ube2f*^*fl*/*fl*^ mice would affect the transcript signature of T_reg_ cells, we generated the inflammation-free female mice, in which one X chromosome expresses wild-type *Foxp3* allele (*Foxp3^wt^*) and the other expresses *Foxp3*^*Cre*^ mutant (designated as *Foxp3*^*Cre*/*wt*^). The random inactivation of one X chromosome in each somatic cell caused about half of T_reg_ cells to express *Foxp3*^*Cre*^ mutant, so *Ube2m* and *Ube2f* genes were deleted in these T_reg_ cells; meanwhile, the other T_reg_ cells expressed normal *Foxp3^wt^* allele and were kept absolutely normal. Due to the protection of the normal T_reg_ cells, the *Foxp3*^*Cre*/*wt*^;*Ube2m*^*fl*/*fl*^;*Ube2f*^*fl*/*fl*^ female mice are inflammation-free and healthy, so the CD4^+^YFP^+^ T_reg_ cells in *Foxp3*^*Cre*/*wt*^;*Ube2m*^*fl*/*fl*^;*Ube2f*^*fl*/*fl*^ mice were deficient of *Ube2m*&*Ube2f* and avoided the suffering from inflammation at the same time.

We then sorted the CD4^+^YFP^+^ T_reg_ cells from *Foxp3*^*Cre*/*wt*^; *Ube2m*^*fl*/*fl*^;*Ube2f*^*fl*/*fl*^ and *Foxp3*^*Cre*/*wt*^ mice, respectively, followed by transcriptome analysis. The *Ube2m*&*Ube2f*-deficient T_reg_ cells (Fig. S2A) showed significant alterations in transcriptome, with 714 genes up-regulated and 1,289 genes down-regulated with fold change (Fc) > 1.5 and *P* value < 0.05 (Fig 2E and Fig. S2, B and C). Among them, many T_reg_ cell function-related genes were down-regulated, including *Il10* [16] and *Cst7* [17] (suppressive cytokines); *Entpd1*, *Nt5e* [18], and *Il2r* [19] (regulators of immune cell metabolism); *Icos* [20], *Ctla4* [21], *Pdcd1* [22], *Lag3* [23], *Nrp1* [24], and *Tigit* [25] (inhibitor via dendritic cells); and *Gzmb* [26] and *Lgals3* [27] (apoptosis inducer) (Fig. 2F). Altered expression in migration-associated surface molecules was also observed in *Ube2m*&*Ube2f*-deficient T_reg_ cells, such as down-regulation of *Cxcr3*, *Cxcr5*, *Cxcr6*, and *Cd44* [28] and up-regulation of *Sell* [29] (Fig. 2F). On the other hand, among the top 20 genes up-regulated in *Ube2m*&*Ube2f*-deficienct T_reg_ cells (Fig. S2D), none of them are previously known to negatively regulate T_reg_ cells upon induction, which is certainly an interesting subject for future investigation.

Gene set enrichment analysis (GSEA) of the altered genes revealed dramatic changes in multiple pathways upon *Ube2m*&*Ube2f* deficiency (Fig. S2E), specially down-regulation of T_reg_-related pathways, such as cytokine–cytokine receptor interaction [30] (Fig. 2G) and T helper 17 (T_H_17) cell differentiation [31] (Fig. S2F). Some up-regulated pathways were also seen in *Ube2m*&*Ubef*-deficienct T_reg_ cells, including DNA replication and repair, cell cycle, and metabolisms of amino acids tyrosine, phenylalanine, and tryptophan, as well as cellular senescence (Fig. S2E and Fig. 2H). How these pathways, upon double knockout of neddylation E2s, are up-regulated and how they functionally regulate T_reg_ cells are the open and interesting questions for future investigation, particularly for possible involvement of senescence pathway.

### Phenotype and transcriptome comparison between *Foxp3*^Cre^;*Ube2m^fl/fl^* and *Foxp3*^Cre^;*Ube2m*^fl/fl^;*Ube2f*^fl/fl^ mice

A direct life-span comparison between *Foxp3*^*Cre*^;*Ube2m*^*fl*/*fl*^ [15] and *Foxp3*^*Cre*^;*Ube2m*^*fl*/*fl*^;*Ube2f*^*fl*/*fl*^ mice revealed a much shortened life span in double E2-null mice (Fig. 3A), indicating a significant contribution of Ube2f in the maintenance of T_reg_ cells to the overall survival of mice. On the other hand, nondetectable phenotypic change in Ube2f T_reg_-depleted mice [15] indicates that the function of Ube2f in T_reg_ cells is fully compensated by Ube2m. Thus, 2 neddylation E2s are functionally redundant in the regulation of T_reg_ cells. While the main functions of Ube2f in T_reg_ cells are compensated by Ube2m, Ube2f fails to functionally compensate Ube2m.

We next compared the transcriptome alterations between *Ube2m*&*Ube2f*-deficient and *Ube2m*-deficient T_reg_ cells [15], along with the *Foxp3*^*Cre*^ control. We normalized 2 sets of data (each from 3 individual mice, run at 2 different time periods), followed by unsupervised cluster analysis. The results showed that the transcriptional profile patterns were largely comparable in *Foxp3*^*Cre*^ control T_reg_ cells from 2 batches of experiments (Fig. 3B), suggesting a reproducibility of the experiments. The profiling comparison revealed 5 clusters (groups) of genes with altered expressions among 3 groups with an overall greater change seen in *Ube2m*&*Ube2f* double-deficient T_reg_ cells (Fig. 3B).

Among the genes selectively down-regulated in double E2-deficient T_reg_ cells (group 1), many genes are known to be related with functional regulation of T_reg_ cells, including *Ccr4* [32], *Egr2* [33], *Il10rb* [34], *Tccam1* [35], *Runx3* [36], *IL10* [16], *Cd3g* [37], *Tnfrsf4* [38], *Pglyrp1* [39], and *Malt1* [40] (Fig. 3C). Among top 20 genes up-regulated in double E2-deficient T_reg_ cells (group 4) (Fig. S3A), Smad7 is the only one known to be involved in regulation of T_reg_ cells, whose up-regulation by the EZH2-FOXP3-RUNX1 axis inhibited T_reg_ cell differentiation in rheumatoid arthritis [41].

GSEA analysis of the genes specially altered in double-null T_reg_ cells revealed the changes in multiple pathways (Fig. S3B), and some down-regulated pathways are known to be involved in T_reg_ cell regulation, such as cytokine–cytokine receptor interaction [30] (Fig. 3D) and T_H_17 cell differentiation [31] (Fig. S3C), whereas lysosome pathway, which is negatively correlated with function of T_reg_ cells [42], along with cell cycle and phosphatidylinositol 3-kinase/Akt pathways, is up-regulated in *Ube2m*&*Ube2f* double-deficient T_reg_ cells (Fig. S3, B and D). Thus, these greater alterations in gene expression are associated with and likely contributed to severer inflammatory disorders in *Foxp3*^*Cre*^;*Ube2m*^*fl*/*fl*^;*Ube2f*^*fl*/*fl*^ mice than in *Foxp3*^*Cre*^;*Ube2m*^*fl*/*fl*^ mice.

### Early-onset fatal inflammatory disorders in *Foxp3*^Cre^;*Rbx1*^fl/fl^;*Sag*^fl/fl^ mice

We next determined potential functional redundancy in T_reg_ cells between 2 E3s, Rbx1 and Sag/Rbx2, dual for both neddylation and ubiquitylation by CRLs. We crossed the *Rbx1-flox*, *Sag-flox*, and *Foxp3*^*YFP*-*Cre*^ (*Foxp3*^*Cre*^) mice [16] to generate the *Foxp3*^*Cre*^;*Rbx1*^*fl*/*fl*^;*Sag*^*fl*/*fl*^ mice, with simultaneous deletion of both *Rbx1* and *Sag*, the only 2 known catalytic subunits of CRLs, in T_reg_ cells. The *Foxp3*^*Cre*^;*Rbx1*^*fl*/*fl*^;*Sag*^*fl*/*fl*^ mice phenocopied the mice ablated of T_reg_ cells in vivo [14], with smaller body size (Fig. 4, A and B), much shortened life span (Fig. 4C), inflammatory changes in multiple organs (Fig. 4D), swollen peripheral immune organs (Fig. 4E and Fig. S4A), and robust activation of immune cells (Fig. 4F and Fig. S7, B and C). Thus, *Rbx1* and *Sag* are absolutely essential for the maintenance of T_reg_ cell fitness.

We then performed the in vivo suppression assay in *Rag1*^*−*/*−*^ immunodeficient mice to confirm the role of *Rbx1*&*Sag* deficiency in the suppressive function of T_reg_ cells and found that the *Rbx1*&*Sag*-deficient T_reg_ cells failed to prevent the colitis induced by T_nai_ cells (Fig. 5A), indicating impaired suppressive function. Thus, despite that the ratios and number of *Rbx1*&*Sag*-deficient T_reg_ cells were dropped dramatically around p20 in vivo (Fig. 5, B to D), the severe inflammation disorder in *Foxp3*^*Cre*^;*Rbx1*^*fl*/*fl*^;*Sag*^*fl*/*fl*^ mice was not the simple consequence caused by the decreased number of T_reg_ cells.

To explore possible underlying mechanism(s) that mediated the function of *Rbx1*&*Sag* in T_reg_ cells, we sorted the CD4^+^YFP^+^ T_reg_ cells from inflammation-free female *Foxp3*^*Cre*/*wt*^;*Rbx1*^*fl*/*fl*^;*Sag*^*fl*/*fl*^ and *Foxp3*^*Cre*/*wt*^ control mice and performed transcription profiling. Deletion of *Rbx1*&*Sag* (Fig. S5A) led to a comprehensive change of the levels of multiple genes in T_reg_ cells, with 641 genes up-regulated and 2,296 genes down-regulated with Fc > 2 and *P* < 0.05 (Fig. 5E and Fig. S5, B and C). Among down-regulated genes, many are reported to be related to the function of T_reg_ cells, including *Il10* [16] and *Cst7* [17] (suppressive cytokines); *Entpd1* and *Nt5e* [18] (regulators of immune cell metabolism); *Icos* [20], *Pdcd1* [22], *Lag3* [23], *Nrp1* [24], and *Tigit* [25] (inhibitor via dendritic cells); and *Lgals3* [27] (apoptosis inducer), and impairment of migration-associated surface molecules, including down-regulation of *Cxcr3*, *Cxcr5*, and *Cd44* [28] (Fig. 5F). Although none of the top 20 up-regulated genes, including *Plk2*, *Sik1*, *Nr4a2*, and *Dusp10* (Fig. S5D), are known to negatively regulate T_reg_ cell functions upon induction, one of the up-regulated genes, *Bach2* (up-regulated with Fc = 1.866 and *P* = 0.013) (Fig. 5G), is known as a negative regulator of T_reg_ cell function [43].

The GSEA pathway analysis of the altered genes (Fc > 1.5 and *P* < 0.05) also revealed the changes of many pathways (Fig. S5E), including down-regulation of T_reg_-related pathways, such as cytokine–cytokine receptor interaction [30] (Fig. 5H), T_H_17 cell differentiation [31] (Fig. S5F), and up-regulation of pathways involving olfactory transduction (Fig. 5I) and metabolisms of retinol and arachidonic acid (Fig. S5E).

### Earlier occurrence of inflammatory disorder in *Foxp3*^Cre^;*Rbx1*^fl/fl^;*Sag*^fl/fl^ mice than in *Foxp3*^Cre^;*Rbx1*^fl/fl^ mice

We next addressed the question of functional redundancy between 2 E3s in T_reg_ cells. Since mice with *Rbx1*-deficient T_reg_ cells already had an early-onset fatal phenotype, whereas mice with *Sag*-deficient T_reg_ cells had no phenotype [15], we first compared the survival of mice with 2 genotypes, *Rbx1&Sag* double deletion versus *Rbx1* single deletion [15], in T_reg_ cells and found no difference, both having early-onset fatal death (Fig. S6A). Moreover, no difference was found in the activation of immune response when mice were near moribund at age of ~20 days (Fig. S6B). We then focused on potential difference in inflammatory responses occurring at the very early stage of 8 days after the birth between 2 types of mice. Compared to the *Foxp3*^*Cre*^ control, the ratio of T_eff/mem_ cells (CD4^+^Foxp3^−^CD44^hi^CD62L^lo^) among T_con_ cells (CD4^+^Foxp3^−^) is elevated dramatically in Rbx1-null or Rbx1&Sag double-null mice (Fig. 6A), indicating a robust immune activation. A substantially higher level was seen in double-null mice (Fig.A and B), demonstrating a more severe immune overactivation in vivo. Thus, like the Ube2m-Ube2f E2 pair, the Rbx1-Sag E3 pair also showed a functional redundancy in T_reg_ cells, although it is minor due to the dominant effect of Rbx1 to mask the Sag effect. Collectively, the Sag function is being fully compensated by Rbx1, whereas the Rbx1 function cannot be compensated by Sag in T_reg_ cells.

### Enhanced transcription alterations caused by *Rbx1*&*Sag* double deficiency in comparison to *Rbx1* single deficiency in T_reg_ cells

We further compared the transcriptome alterations between *Rbx1*&*Sag*- and *Rbx1*-deficient T_reg_ cells, along with *Foxp3*^*Cre*^ control T_reg_ cells, generated previously [15] and in this study, which showed large reproducibility (Fig. 6C, first 6 columns). Unsupervised cluster analysis of the transcriptome data revealed 4 groups of alterations: (a) genes down-regulated in both *Rbx1*&*Sag* double-deficient and *Rbx1* single-deficient T_reg_ cells; (b) genes selectively down-regulated in *Rbx1*&*Sag* double-deficient T_reg_ cells; (c) genes selectively up-regulated in *Rbx1*&*Sag* double-deficient T_reg_ cells; and (d) genes up-regulated in both *Rbx1*&*Sag* double-deficient and *Rbx1* single-deficient T_reg_ cells (Fig. 6C). Many group 2 genes are related with T_reg_ cell regulation, including *Cxcr3* [44], *Itch* [45], *Il7rb* [46], and *Il10rb* [34] (Fig. 6D). Among the top 20 up-regulated genes in group 3 (Fig. S7A), none of them are known to negatively regulate T_reg_ cell function upon induction, and *Bach2* in group 3 (not in the list of top 20 genes) appears to be the only gene known as a negative regulator of T_reg_ cells [43] (Fig. 6E).

GSEA analysis of the genes specially altered in *Rbx1*&*Sag*-deficient T_reg_ cells revealed changes of multiple pathways (Fig. S7B), where cytokine–cytokine receptor interaction was down-regulated (Fig. 6F), which are involved in T_reg_ cell regulation [30] and up-regulation of multiple pathways, such as the olfactory transduction pathway (Fig. 6G). Taken together, it appears that many genes are associated with and may contribute to severer inflammatory disorder seen in *Foxp3*^*Cre*^;*Rbx1*^*fl*/*fl*^;*Sag*^*fl*/*fl*^ mice.

### Phenotypes and transcriptome comparisons of mice and T_reg_ cells with double E2 versus double E3 deficiency

It is well established that cullin neddylation is essential for activation of CRLs, and Rbx1 and Sag serve as dual E3 for both neddylation and CRL-medicated ubiquitylation [47,48]. To verify the functional relationship between neddylation and CRLs in T_reg_ cells, we compare the degree of autoimmune disorders and transcriptional alterations caused by *Ube2m*&*Ube2f* versus *Rbx1*&*Sag* deficiency in T_reg_ cells.

Although both *Foxp3*^*Cre*^;*Ube2m*^*fl*/*fl*^;*Ube2f*^*fl*/*fl*^ mice and *Foxp3*^*Cre*^; *Rbx1*^*fl*/*fl*^;*Sag*^*fl*/*fl*^ mice suffered from severe autoimmune disorders with much shortened life span, the degree of severity still differs. In fact, the survival of *Foxp3*^*Cre*^;*Rbx1*^*fl*/*fl*^;*Sag*^*fl*/*fl*^ mice is significantly shorter than that of *Foxp3*^*Cre*^;*Ube2m*^*fl*/*fl*^;*Ube2f*^*fl*/*fl*^ mice, with *P* = 0.0350 (Fig. 7A). In the peripheral lymph nodes from mice at p19 to p21, the ratios of T_eff/mem_ cells (CD4^+^Foxp3^−^CD44^hi^CD62L^lo^) among T_con_ cells (CD4^+^Foxp3^−^) are higher in *Foxp3*^*Cre*^;*Rbx1*^*fl*/*fl*^;*Sag*^*fl*/*fl*^ mice than in *Foxp3*^*Cre*^; *Ube2m*^*fl*/*fl*^; *Ube2f*^*fl*/*fl*^ mice, although the difference is not statistically significant due to variations among mice (Fig. 7B). Thus, double E3-null mice had more dramatic immune overactivation than double E2-null mice.

The comparison of transcriptome data of CD4^+^YFP^+^ T_reg_ cells from *Foxp3*^*Cre*/*wt*^, *Foxp3*^*Cre*/*wt*^;*Ube2m*^*fl*/*fl*^;*Ube2f*^*fl*/*fl*^, and *Foxp3*^*Cre*/*wt*^;*Rbx1*^*fl*/*fl*^;*Sag*^*fl*/*fl*^ mice revealed 6 groups of altered genes (up or down), unique to either double E2-deficient or double E3-deficient or both deficient T_reg_ cells (Fig. 7C). We mainly focused on genes uniquely altered in double E3-null or double E2-null T_reg_ cells. Among selectively down-regulated genes in double E3-null T_reg_ cells, many are related to function or regulation of T_reg_ cells (Fig. 7D), whereas among up-regulated genes (Fig. S8A), none of them are known to negatively regulate T_reg_ cells upon induction. Similarly, among selectively altered genes (down and up) in double E2-null T_reg_ cells (Fig. S9, A and B), few down-regulated, but not up-regulated, genes were known to regulate T_reg_ cell functions. Nevertheless, the changes unique in double E3-null T_reg_ cells likely represent CRL-dependent mechanism, whereas the changes unique in double E2-null T_reg_ cells likely represent CRL-independent, but neddylation-dependent, mechanisms, consistent with our recent studies [49–51].

Similar GSEA pathway analysis of 2 groups of genes unique to double E3-deficient T_reg_ cells revealed remarkable alterations in many pathways (Fig. S8B), including down-regulation of Hippo signaling pathway (Fig. 7E), which plays an important role in the maintenance of T_reg_ cell function [52], and of pathways related to lysosome, as well as up-regulation of olfactory transduction and metabolisms of arachidonic acid and retinol (Fig. S8, B and C), which are previously unknown in regulation of the T_reg_ cell functions.

GSEA pathway analysis of 2 groups of genes unique to double E2-deficient T_reg_ cells also revealed alterations in a number of pathways (Fig. S9C), including down-regulation of ubiquitin-mediated proteolysis and up-regulation of DNA replication pathways (Fig. S9, D and E). Notably, the number of genes unique to double E2-deficient T_reg_ cells is relatively smaller than in double E3-deficient T_reg_ cells, which is consistent with the observation that *Foxp3*^*Cre*^;*Rbx1*^*fl*/*fl*^;*Sag*^*fl*/*fl*^ mice suffer more dramatic autoimmune disorders than do the *Foxp3*^*Cre*^;*Ube2m*^*fl*/*fl*^;*Ube2f*^*fl*/*fl*^ mice.

Collectively, the early-onset fatal disorders seen in both double E2- and double E3-deficient mice indicate functional similarity of neddylation and CRLs in regulation of T_reg_ cells. More severe inflammatory disorders in double E3-deficient mice suggest a neddylation-independent function of CRLs in T_reg_ cells.

## Discussion

In this study, we investigated the role of the neddylation-CRL axis in functional regulation of T_reg_ cells using several in vivo conditional knockout mouse models. Double deletion of neddylation E2s *Ube2m*&*Ube2f* or E3s *Rbx1*&*Sag* in T_reg_ cells leads to the impairment of suppressive function of T_reg_ cells and results in severe autoimmune disorders, fully demonstrating the pivotal role of the neddylation-CRL axis in the maintenance of T_reg_ cell fitness. Thus, the neddylation-CRL axis is essential for T_reg_ cell functions as the new key regulators of T_reg_ cells beyond Foxp3 [11–13].

The functional independence of the Ube2m-Rbx1 and Ube2f-Sag axes was previously demonstrated both biochemically [4,53] and biologically [8,9]. Specifically, our recent study revealed that the Ube2m-Rbx1 axis plays a pivotal role in functional regulation of T_reg_ cells, while the Ube2f-Sag axis is dispensable in T_reg_ cells at the steady status [15]. This T_reg_-based functional study also supports the notion of functional independence between the Ube2m-Rbx1 and Ube2f-Sag axes.

Here, we used double knockout of either neddylation E2s or E3s in T_reg_ cells and showed that both E2s and E3s are functionally redundant in T_reg_ cells, as evidenced by the following: (a) The phenotypes of autoimmune disorders in *Foxp3*^*Cre*^;*Ube2m*^*fl*/*fl*^;*Ube2f*^*fl*/*fl*^ (double E2 knockout in T_reg_ cells) mice are much more severe than in *Foxp3*^*Cre*^;*Ube2m*^*fl*/*fl*^ (single E2 knockout in T_reg_ cells) mice with much shortened life span (Fig. 3A). (b) The same autoimmune phenotypes are severer in *Foxp3*^*Cre*^;*Rbx1*^*fl*/*fl*^;*Sag*^*fl*/*fl*^ (double E3s knockout in T_reg_ cells) mice than in *Foxp3*^*Cre*^;*Rbx1*^*fl*/*fl*^ (single E3 knockout in T_reg_ cells) mice at the very early age only (Fig. 6A and B). We further found that the autoimmune phenotypes were much severer in *Foxp3*^*Cre*^;*Rbx1*^*fl*/*fl*^;*Sag*^*fl*/*fl*^ mice than in *Foxp3*^*Cre*^;*Ube2m*^*fl*/*fl*^;*Ube2f*^*fl*/*fl*^ mice (Fig. 7A).

We conclude from these results that (a) the Ube2f-Sag axis plays a role in T_reg_ cells, which is, however, compensated by the Ube2m-Rbx1 axis. More specifically, the autoimmune phenotypes by Ube2f depletion were largely compensated by Ube2m, whereas the same autoimmune phenotypes by Sag depletion were minimally compensated by Rbx1, due to the dominant role of Rbx1 in T_reg_ cells. (b) The functional redundancy in T_reg_ cells is in one-way direction from the Ube2m-Rbx1 axis to the Ube2f-Sag axis, indicating much more significant role of CRL1 to CRL4 than of CRL5. (c) Rbx1 and Sag play neddylation-dependent and neddylation-independent roles in regulating T_reg_ cell fitness.

To pursue the possible underlying mechanism, we performed the comprehensive transcriptome profiling analyses of T_reg_ cells derived from the wild-type control or double knockout mice, and compared between single-null versus double-null T_reg_ cells for both E2s or E3s, respectively. Compared to the wild-type control T_reg_ cells, T_reg_ cell deficiency of *Ube2m*&*Ube2f* caused dramatic alterations in mRNA transcriptome. Many genes (such as *Il10*, *Cst7*, and *Cxcr3* to *Cxcr6*) and pathways (e.g., cytokine–cytokine receptor interaction and T_H_17 cell differentiation), previously known to regulate T_reg_ cells, were down-regulated (Fig. 2 and Fig. S2), supporting the lost-of-function phenotypes. On the other hand, interestingly, many up-regulated genes (none of the top 20 genes) (Fig. S2D), with the exception of *Sell* (Fig. 2F) [29], or pathways (Fig. S2E) are previously unknown to negatively regulate T_reg_ cell function upon activation. Nevertheless, some up-regulated pathways, such as cellular senescence (Fig. 2H), could contribute to the loss-of-function phenotype and deserve detailed future investigation.

Likewise, compared to the wild-type control T_reg_ cells, T_reg_ cell deficiency of *Rbx1*&*Sag* also caused dramatic alterations in mRNA transcriptome. Many genes (such as *Il10*, *Cst7*, *Entpd1*, and *Nt5e*) and pathways (again cytokine–cytokine receptor interaction and T_H_17 cell differentiation), previously known to regulate T_reg_ cells, were down-regulated (Fig. 5 and Fig. S5), further supporting the lost-of-function phenotypes. On the other hand, interestingly, many up-regulated genes (again none of the top 20 genes) or pathways are previously unknown to regulate T_reg_ cell function upon activation. One exception is *Bach2*, a known negative regulator of T_reg_ cell functions [43], which is up-regulated in *Rbx1*&*Sag*-deficienct T_reg_ cells (Fig. 5G). However, it is completely unknown how some up-regulated pathways, such as those involving olfactory transduction (Fig. 5I and Fig. S5E), negatively regulate T_reg_ cell functions.

Transcriptome comparison between T_reg_ cell deficiency of *Ube2m*&*Ube2f* versus *Ube2m* also showed many alterations. A list of 11 genes (e.g., *Ccr4*, *Egr2*, and *Il10*), uniquely down-regulated in double E2 null (Fig. 3C), are all known to regulate T_reg_ cell functions. Again, the pathways associated with cytokine–cytokine receptor interaction and T_H_17 cell differentiation were also down-regulated uniquely to double E2 null T_reg_ cells (Fig. 3D and Fig. S3C). Among top 20 up-regulated genes (Fig. S3A), Smad7 is the only one reported to inhibit T_reg_ cell differentiation in rheumatoid arthritis upon up-regulation by the EZH2-FOXP3-RUNX1 axis [41]. The other up-regulated genes, along with up-regulated pathways (Fig. S3B), unknown to negatively regulate T_reg_ cell functions, open a new window to study neddylation involvement in T_reg_ cell functions, for example, how up-regulation of lysosome pathway (Fig. S3B and D) contributes to the loss-of-function phenotype of T_reg_ cells.

Transcriptome comparison between T_reg_ cell deficiency of *Rbx1*&*Sag* versus *Rbx1* also showed many alterations, but to a lesser extent (Fig. 6C and Fig. S7). Down-regulation of *Cxcr3*, *Fas*, *Egr2*, and *Itch* and of cytokine–cytokine receptor interaction pathway appeared to be unique to double E3-null T_reg_ cells (Fig. 6, D and F). Among up-regulated genes and pathways (Fig. S7), *Bach2* and olfactory transduction (Fig. 6, E and G) appeared to be unique. Given a minor phenotypic difference between Rbx1/Sag double-null and Rbx1 single-null mice, these alterations are unlikely to play a major role in the loss-of-function phenotypes.

Finally, we compared the phenotypes of *Foxp3*^*Cre*^;*Ube2m*^*fl*/*fl*^; *Ube2f*^*fl*/*fl*^ (double E2-null) mice with *Foxp3*^*Cre*^;*Rbx1*^*fl*/*fl*^;*Sag*^*fl*/*fl*^ (double E3-null) mice and found that double E3-null mice suffer more severe autoimmune disorders with a shorter life span (Fig. 7A). Consistently, the transcriptional alterations caused by *Rbx1*&*Sag* deficiency in T_reg_ cells are more dramatic than those in *Ube2m*&*Ube2f*-deficient T_reg_ cells, with a more remarkable decrease of T_reg_ cell regulatory genes and down-regulation of Hippo signaling pathway (Fig. 7, D and E), which was known to play an important role in the maintenance of T_reg_ cell function [52]. Among up-regulated genes and pathways, such as olfactory transduction and metabolisms of retinol and arachidonic acid, which are unique to double E3-null (Fig. S8B and C), it is completely unknown whether and how they negatively regulate T_reg_ cell function, nor their contribution to severe phenotypes. In addition, we also identified genes and pathways unique to double E2 knockout T_reg_ cells (Fig. 7C and Fig. S9). These alterations are likely independent of inactivation of Rbx1/Sag-CRLs but involve neddylation modification of non-cullin substrates via other dual E3s for both neddylation and ubiquitylation [49–51].

It is not surprising that many changes in gene expression occur in these T_reg_ conditional knockout models, given the fact that neddylation E2/E3s are required for cullin neddylation to activate CRLs, which are responsible for ubiquitylation and degradation of ~20% cellular proteins doomed for proteasome degradation [54], thus regulating many biochemical and biological processes. Mechanistically, it is anticipated that inactivation of CRLs, resulting from depletion of neddylation E2/E3, will cause the accumulation of many cellular substrates, including (a) transcription factors and repressors, which would directly affect gene expression, and (b) other signal molecules and cell cycle regulators, which would indirectly affect gene expression. Unfortunately, given that T_reg_ cells are a rare population in vivo and no in vitro cell lines were established, it is technically challenging to define the detailed underlying mechanisms by these altered genes and pathways in our T_reg_ cell conditional knockout setting. Nevertheless, our study defined genes and pathways responsible for both neddylation-dependent and neddylation-independent roles for neddylation E2s and dual E3s.

In summary, in this study, we used mouse models of T_reg_ cell double knockout of neddylation E2s or E3s and made 3 major findings: (a) Neddylation enzymes play an absolute essential role in the maintenance of T_reg_ cell fitness and their dual disruption causes severer autoimmune phenotypes; (b) there is previously unknown functional redundancy between Ube2m and Ube2f, and to a lesser extent between Rbx1 and Sag; and (c) dual E3s Rbx1 and Sag have neddylation-dependent and neddylation-independent role. Finally, our study has sound physiological and pathological relevance to autoimmune diseases, given the fact that a variety of human autoimmune diseases, including systemic lupus erythematosus, inflammatory bowel disease, and rheumatoid arthritis, are subjected to fine regulation by CRLs [55], whose activation requires neddylation E2, UBE2M/UBE2F, and E3 RBX1/RBX2.

## Materials and Methods

### Mice

The *Foxp3YFP*-*Cre* mice (The Jackson Laboratory, no. 016959) were provided by X. Feng (Peking Union Medical College, China) [56]. The *Ube2m*-*flox*, *Ube2f*-*flox*, *Rbx1*-*flox*, and *Sag*-*flox* mice were generated previously [15,57]. The *Rag1*^*−*/*−*^ mice were obtained from GemPharmatech Co. Ltd., China (strain no. T004753).

Mice were fed in specific pathogen-free (SPF) conditions. All animal experiments were approved by the Animal Ethics Committee of Zhejiang University; animal care was provided in accordance with the principles and procedures by the regulatory standards at Zhejiang University Laboratory Animal Center.

### Flow cytometry

Peripheral lymph nodes and spleens from indicated mice were grinded into single cells. Cells were washed in phosphate-buffered saline containing 2% (w/v) fetal bovine serum and then stained with indicated antibodies for analysis of surface proteins. For intracellular proteins, cells were fixed and permeabilized with Pharmingen Transcription Factor Buffer Set (BD Pharmingen, 562574). Flow cytometry was performed on CytoFLEX LX (Beckman).

Antibodies used were listed as follows: anti-CD4 (GK1.5, eBioscience), anti-CD8α (53-6.7, BD Pharmingen), anti-CD44 (IM7, BioLegend), anti-CD62L (MEL-14, BioLegend), and anti-Foxp3 (150D, BioLegend).

### Cell sorting

The CD4^+^ T cells were isolated from the single-cell suspension of peripheral lymph nodes and spleens by Mouse CD4 T Lymphocyte Enrichment Set-DM (BD Biosciences, 558131), followed by fluorescence-activated cell sorting to purify T_reg_ cells (CD4^+^YFP^+^) and/or T_nai_ cells (CD4^+^YFP^−^CD44^lo^CD62L^hi^) with purities >99%, performed on SONY Cell Sorter (SH800S).

### In vivo suppression assay

T_nai_ cells (CD4^+^YFP^−^CD44^lo^CD62L^hi^, 4 × 10^5^) and T_reg_ cells (CD4^+^YFP^+^, 2 × 10^5^), sorted from the peripheral lymph nodes and spleens of indicated mice (8 to 12 weeks old), were administrated into *Rag1*^−/−^ mice via intraperitoneal injection as described [58]. Four weeks after the intraperitoneal injection, mouse colons were harvested and fixed by formalin, followed by H&E staining.

### Transcriptome profiling

CD4^+^YFP^+^ T_reg_ cells were sorted from the peripheral lymph nodes and spleens of indicated mice (8 to 12 weeks old). To generate enough materials, 2 to 3 mice were pooled for one sample. RNA was purified from the sorted cells with the miRNeasy Mini Kit (Qiagen, 21704). The RNAs were reverse-transcribed, amplified, and labeled (Affymetrix GeneChip Pico Kit, 703308) to achieve enough cDNAs. Then, the cDNA samples were hybridized to Clariom S Arrays, mouse (902931). The Applied Biosystems Expression Console Software 1.4 was employed to analyze the microarray datasets.

### Statistical analysis

The *P* values were calculated by Mann–Whitney test, 2-tailed unpaired Student’s *t* test, using GraphPad Prism software. Mouse survival and respective *P* values were analyzed by the log-rank test. The statistical significance was evaluated by *P* < 0.05. All error bars represent SEM (*n* ≥ 3).

## Data Availability

The microarray data generated in this study have been deposited in Gene Expression Omnibus under accession code GSE237499.
