# Supplementary material for: The Functional Redundancy of Neddylation E2s and E3s in Modulating the Fitness of Regulatory T Cells
Source: Research (Wash D C). 2023 Aug 18;6:0212. doi: 10.34133/research.0212 (PMC10437198; doi:10.34133/research.0212)
Supplement: Supplementary 1 — Figs. S1 to S9 [file research.0212.f1.pdf]

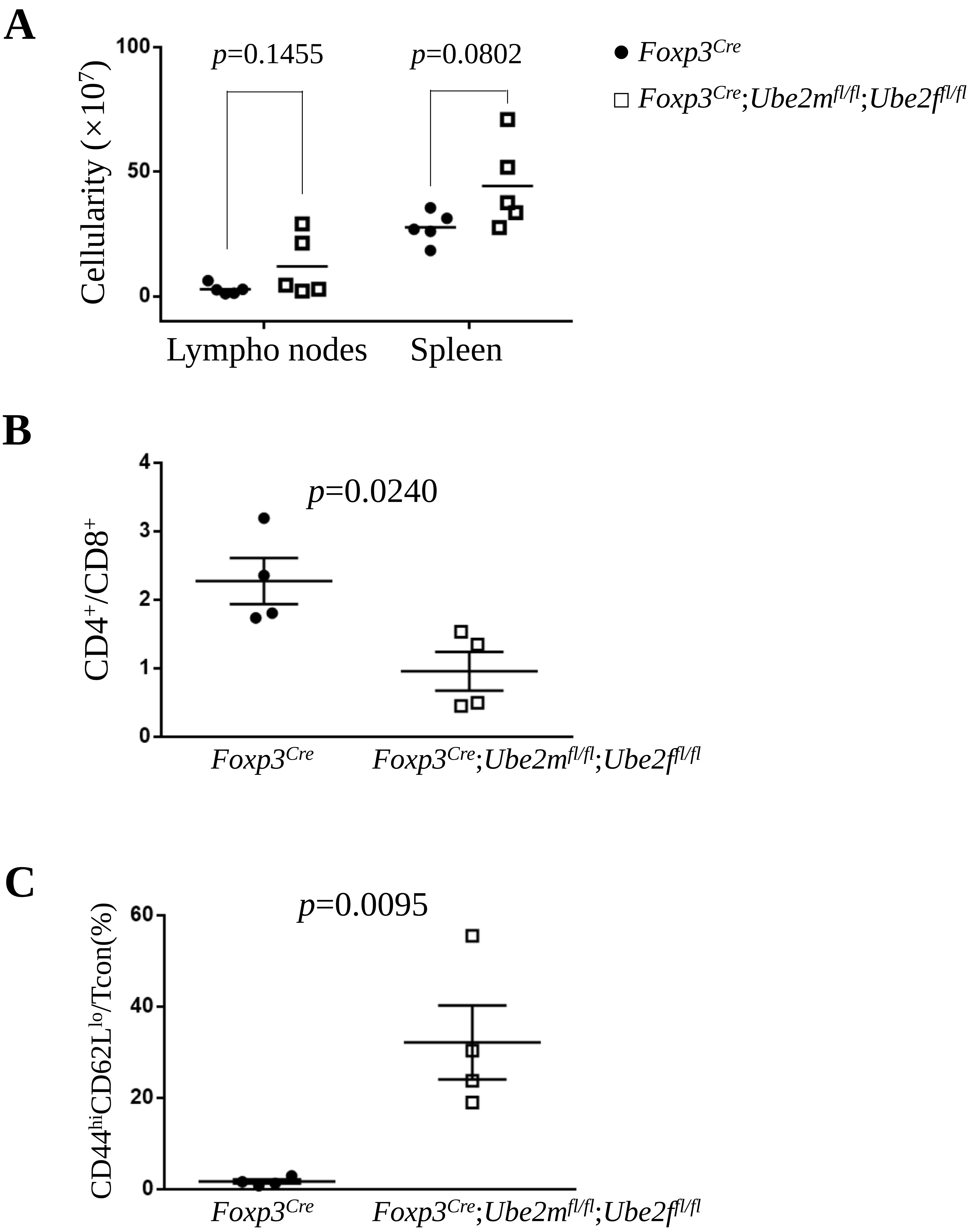

Fig. S1. Auto-immune disorders in *Foxp3^{Cre};Ube2m$^{fl/fl}$;Ube2f$^{fl/fl}$* mice

(A) Total cell numbers in peripheral lymph nodes and spleen from *Foxp3^{Cre}* and *Foxp3^{Cre};Ube2m$^{fl/fl}$;Ube2f$^{fl/fl}$* mice (p19-21, *n*=5).

(B) $CD4^+/CD8^+$ ratios in peripheral lymph nodes from *Foxp3^{Cre}* and *Foxp3^{Cre};Ube2m$^{fl/fl}$;Ube2f$^{fl/fl}$* mice (p19-21, *n*=5).

(C) The proportion of $CD44^{hi}CD62L^{lo}$ effector/memory cells among Tcon cells in peripheral lymph nodes from *Foxp3^{Cre}* and *Foxp3^{Cre};Ube2m$^{fl/fl}$;Ube2f$^{fl/fl}$* mice (p19-21, *n*=5).

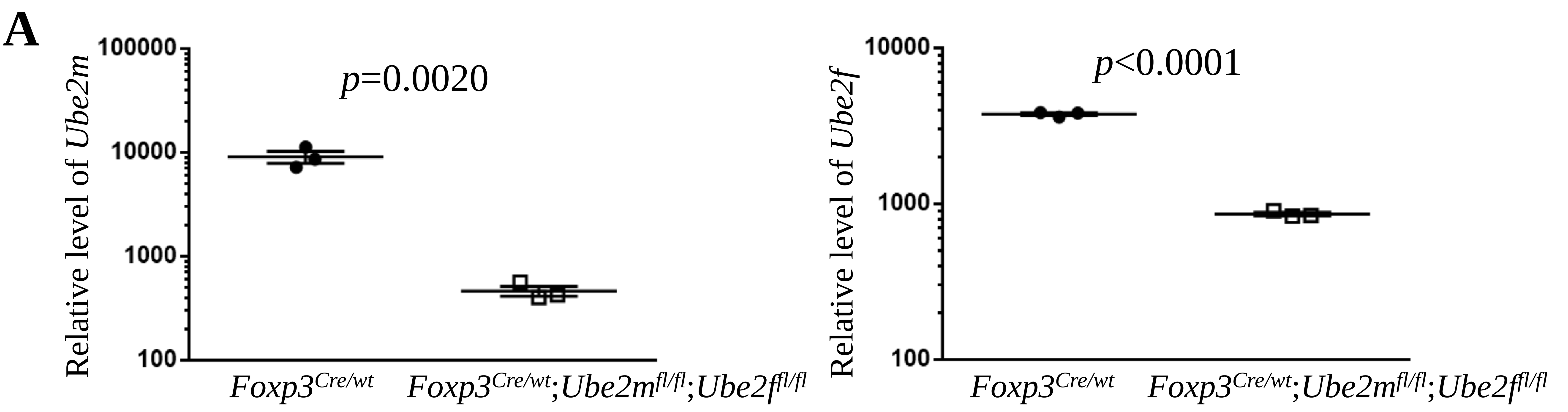

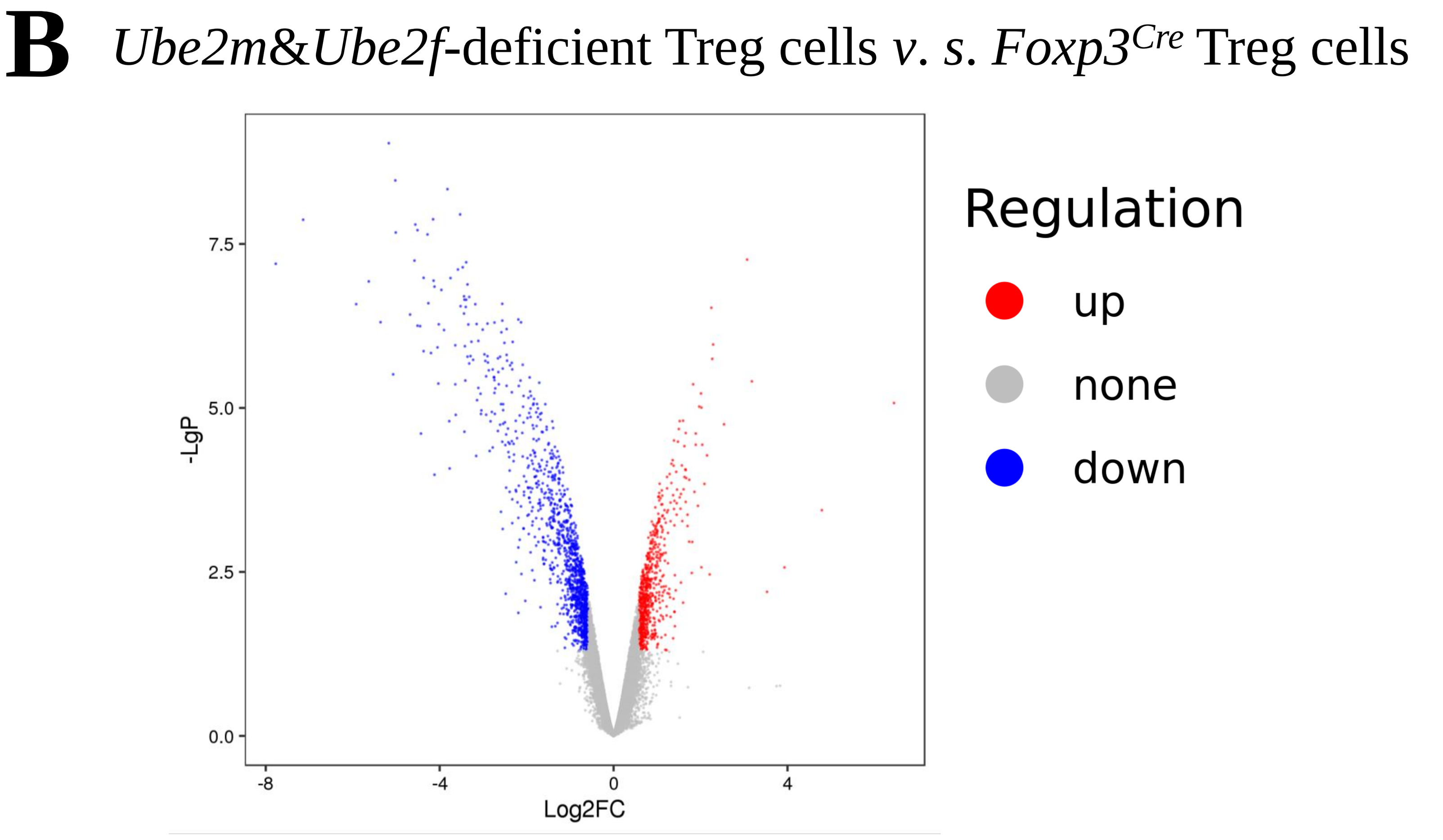

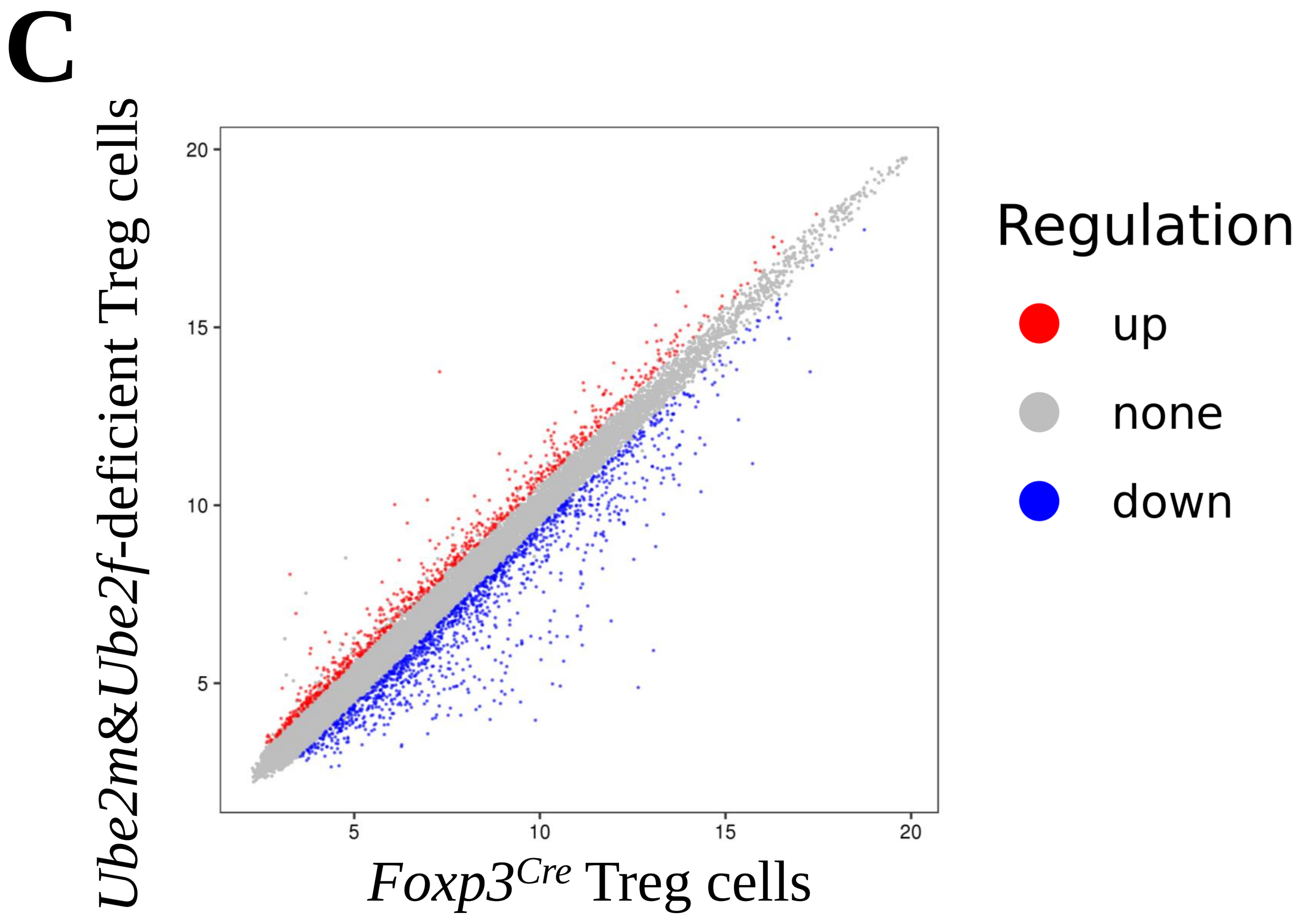

D

Gene symbol	*Foxp3*$^{Cre/wt}$ (average, log2)	*Foxp3*$^{Cre/wt}$;*Ube2m*$^{fl/fl}$;*Ube2f*$^{fl/fl}$ (average, log2)	Fold change
Gm11096	7.30	13.75	87.427
Ccl22	3.27	8.06	27.665
Jchain	6.09	10.02	15.242
Fscn1	3.43	6.96	11.551
Pde3b	6.97	10.15	9.063
Chil5	6.43	9.50	8.398
Itih5	8.91	11.45	5.816
Satb1	13.71	16.0	4.891
1700025G04Rik	11.17	13.44	4.823
Cnn3	6.21	8.46	4.757
H2-M2	4.22	6.43	4.627
Ntn4	3.83	5.98	4.438
Adcy6	5.75	7.84	4.257
Parp8	11.19	13.23	4.112
Atp1b1	8.24	10.26	4.056
Apol7c	3.81	5.83	4.056
Actn1	11.99	14.0	4.028
Als2cl	7.04	9.01	3.918
Ly6c1	13.12	15.06	3.837
Pde4d	10.41	12.3	3.706

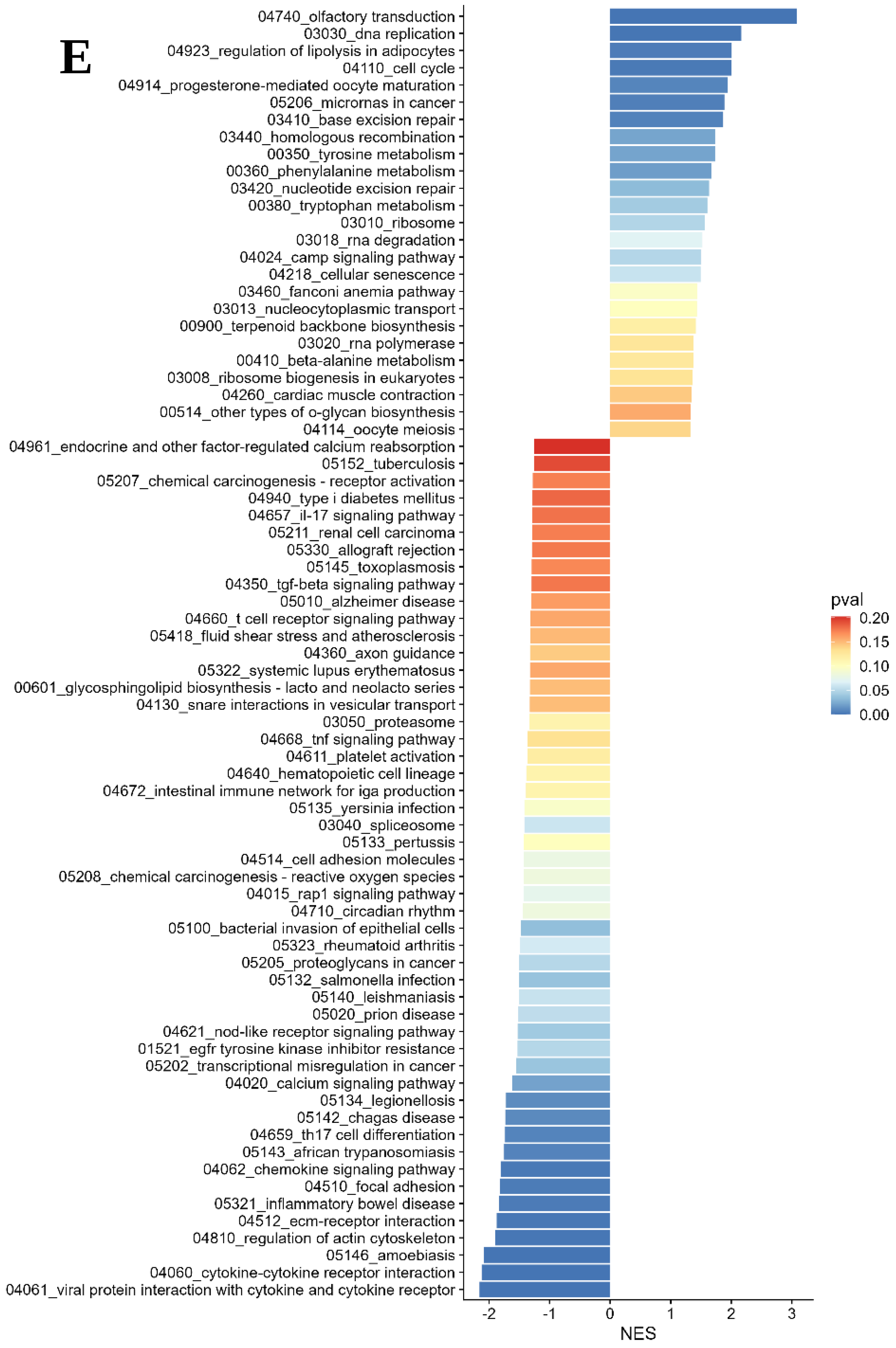

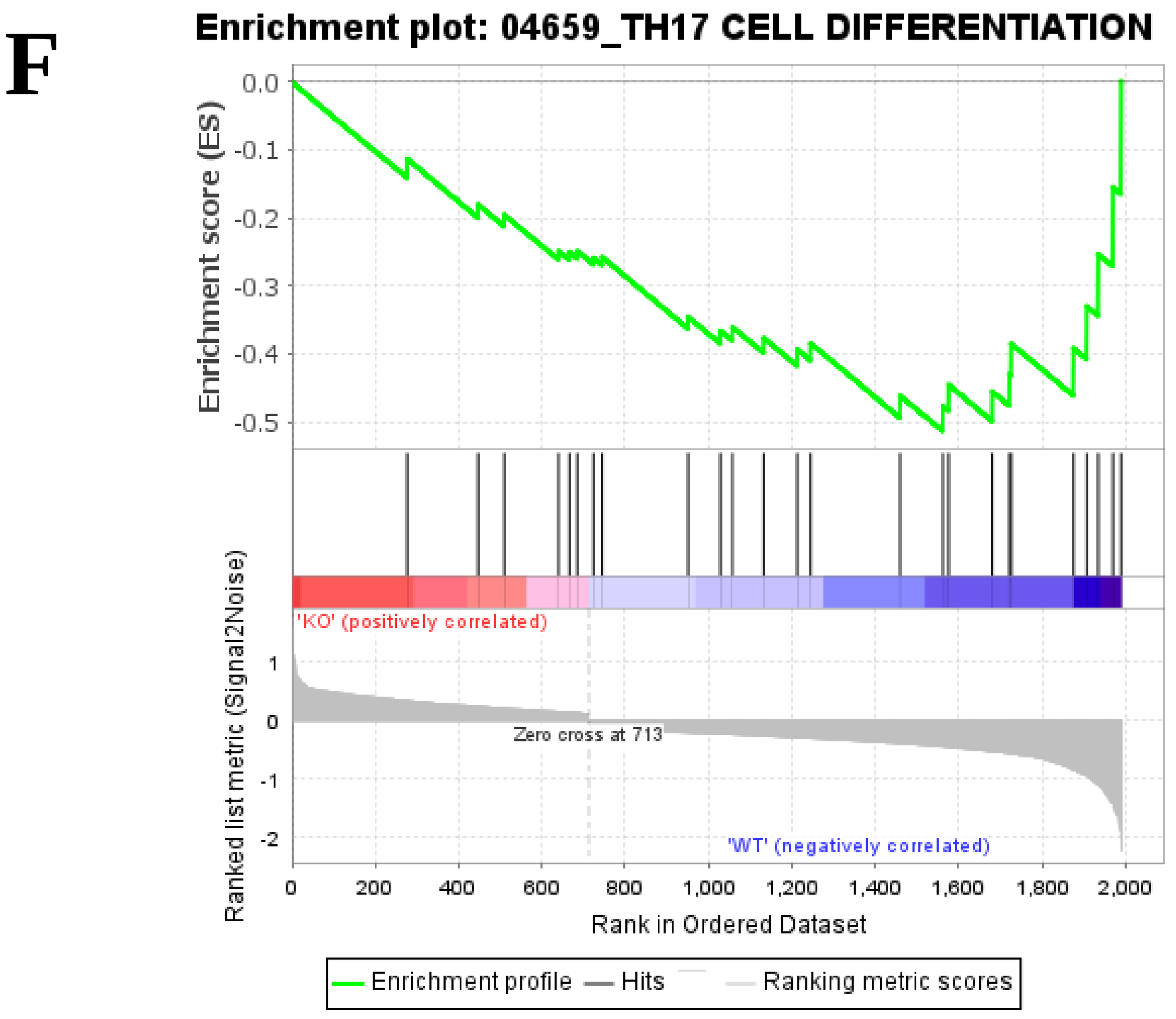

Fig. S2. Transcriptional alterations in *Ube2m&Ube2f*-deficient Treg cells

(A) Transcriptional profiling revealed dramatic decreased levels of *Ube2m* and *Ube2f* in *Ube2m&Ube2f*-deficient Treg cells.

(B) Volcano plot of the transcriptional alterations in *Ube2m&Ube2f*-deficient Treg cells compared to the $Foxp3^{Cre}$ control Treg cells.

(C) Scatter plot of the transcriptional programs revealed from *Ube2m&Ube2f*-defificient Treg cells compared to the $Foxp3^{Cre}$ control Treg cells.

(D) Top 20 up-regulated genes in *Ube2m&Ube2f*-deficient Treg cells compared to the $Foxp3^{Cre}$ control Treg cells with alters more than 1.5-folds and *p*-values<0.05.

(E) GSEA pathway analysis of the transcriptional alterations in *Ube2m&Ube2f*-deficient Treg cells compared to the $Foxp3^{Cre}$ control Treg cells; genes with alterations more than 1.5-folds, with *p*-values<0.05 were selected.

(F) Gene set enrichment analysis of T_H17 cell differentiation genes in $CD4^+YFP^+$ Treg cells from $Foxp3^{Cre/wt}$ and $Foxp3^{Cre/wt};Ube2m^{fl/fl};Ube2f^{fl/fl}$ mice, determined by transcriptional profiling with Fc>1.5 and $p<0.05$.

A

Gene symbol	*Foxp3*$^{Cre/wt}$ (average, log2)	*Foxp3*$^{Cre/wt}$;*Ube2m*$^{fl/fl}$;*Ube2f*$^{fl/fl}$ (average, log2)	Fold change
Gm3706	5.98	8.29	4.946
Gm8127	5.96	7.86	3.715
Gm3123	4.77	6.59	3.529
Spi1	4.93	6.70	3.400
Vmn2r96	8.01	9.45	2.732
Rapgef4	7.01	8.40	2.626
Gm7980	4.84	6.18	2.528
Vmn1r114	5.61	6.94	2.507
Gm10922	4.97	6.24	2.417
Ubap2	10.63	11.81	2.271
Klf7	9.62	10.79	2.261
St8sia1	10.79	11.95	2.227
Bub1b	5.32	6.48	2.223
Gm2046	6.30	7.43	2.193
Dgke	5.61	6.73	2.175
Hells	6.54	7.65	2.170
Pcdh7	3.53	4.65	2.170
Adck5	6.18	7.28	2.138
Smad7	9.82	10.90	2.126
Armcx1	4.03	5.09	2.084

B

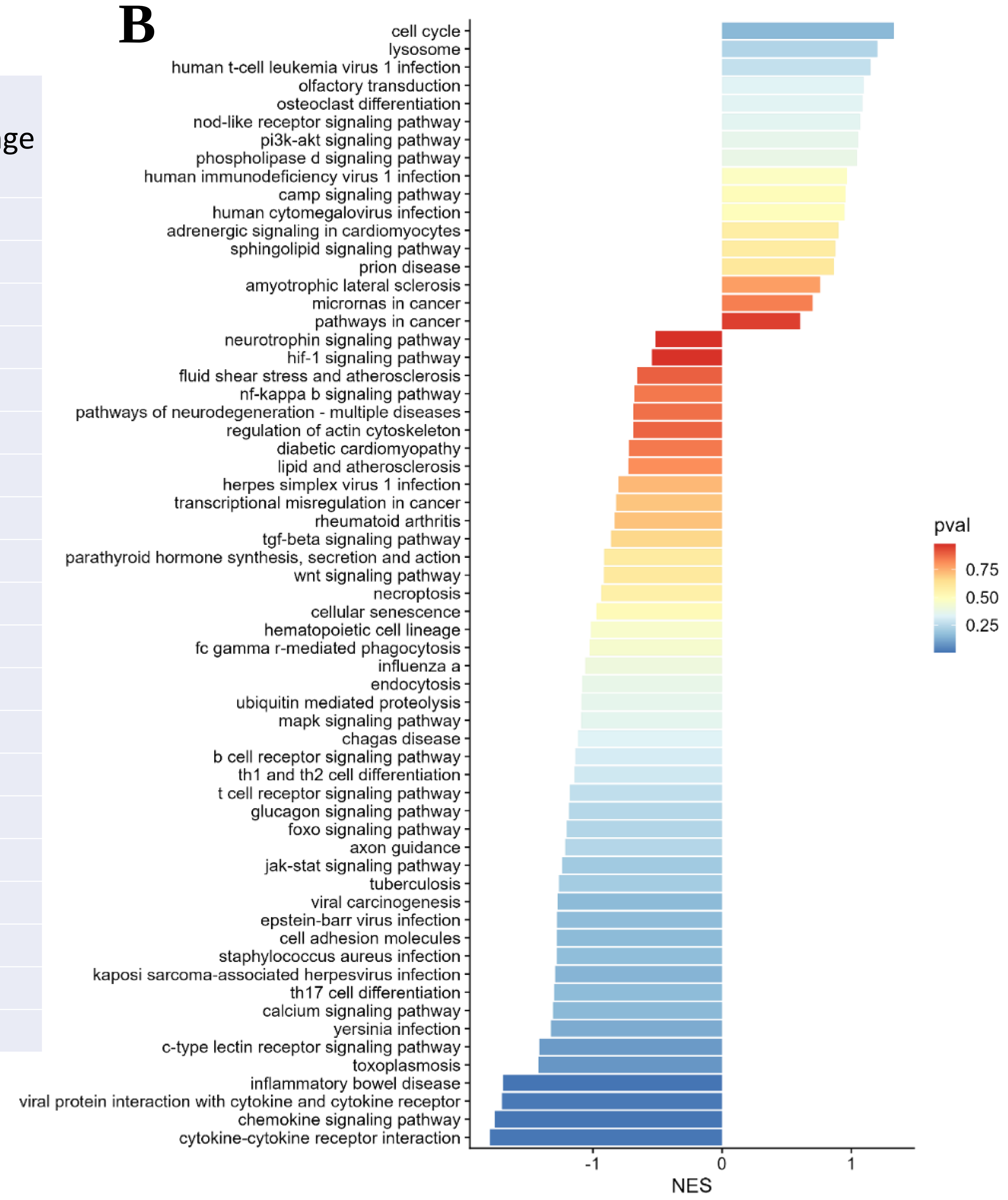

C

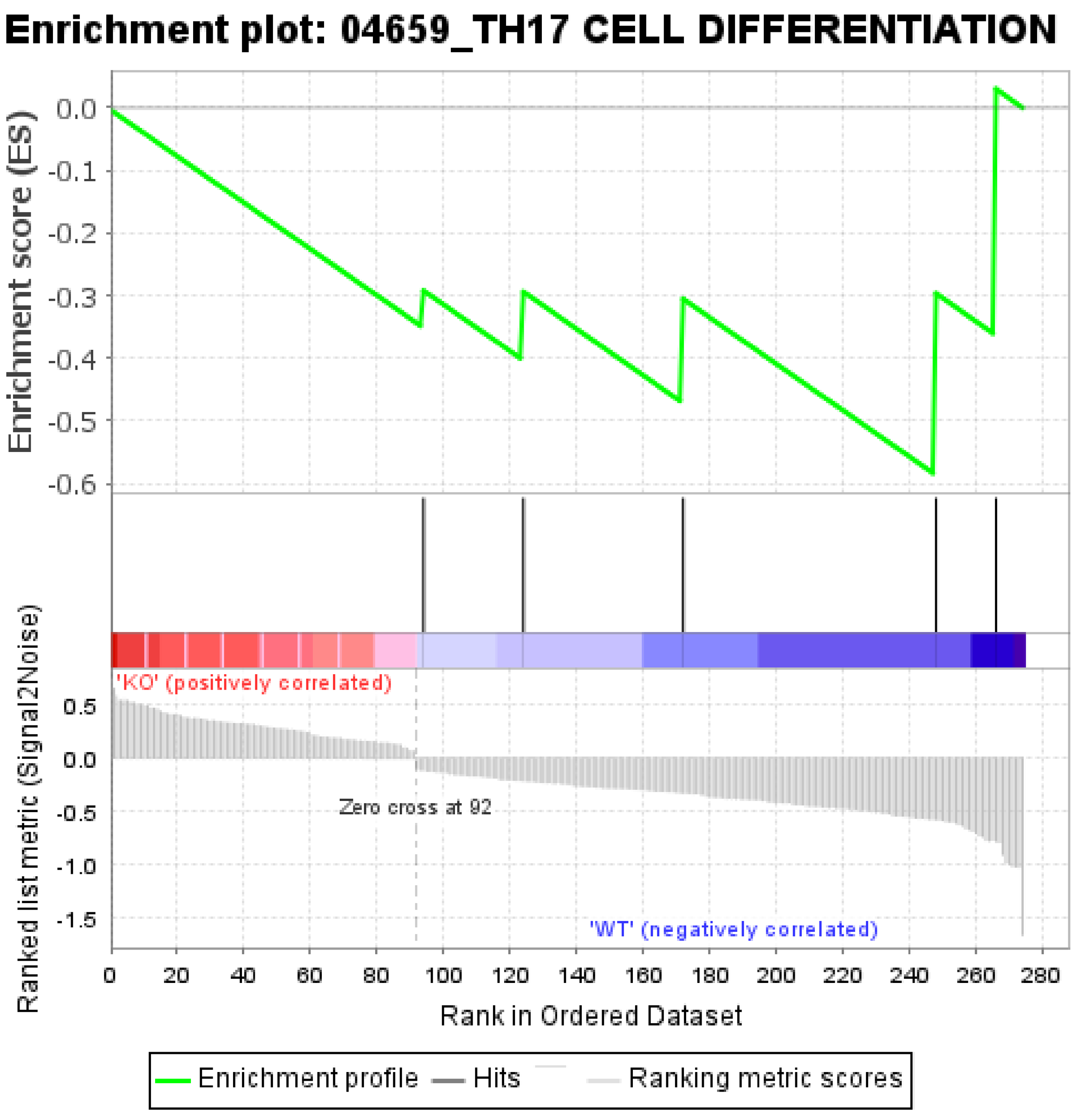

D

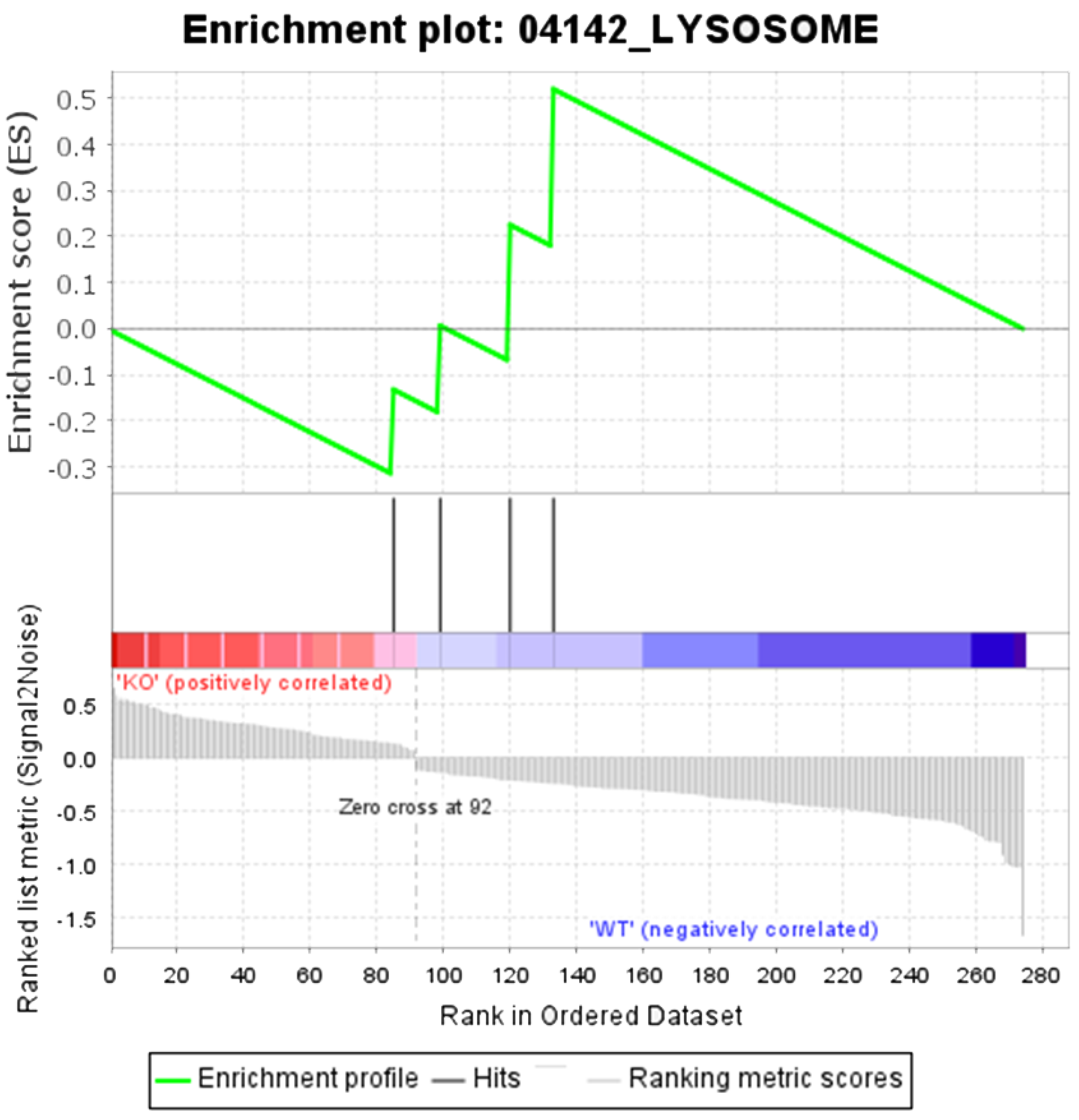

Fig. S3. GSEA pathway analysis of genes altered in *Ube2m&Ube2f*-deficient, but not *Ube2m*-deficient Treg cells

(A) Top 20 up-regulated genes in *Ube2m&Ube2f*-deficient, but not *Ube2m*-deficient, Treg cells.

(B) GSEA pathway analysis of genes altered in *Ube2m&Ube2f*-deficient, but not *Ube2m*-deficient Treg cells.

(C) Gene set enrichment analysis of T_H17 cell differentiation genes in genes altered in *Ube2m&Ube2f*-deficient, but not *Ube2m*-deficient Treg cells.

(D) Gene set enrichment analysis of lysosome genes in genes altered in *Ube2m&Ube2f*-deficient, but not *Ube2m*-deficient Treg cells.

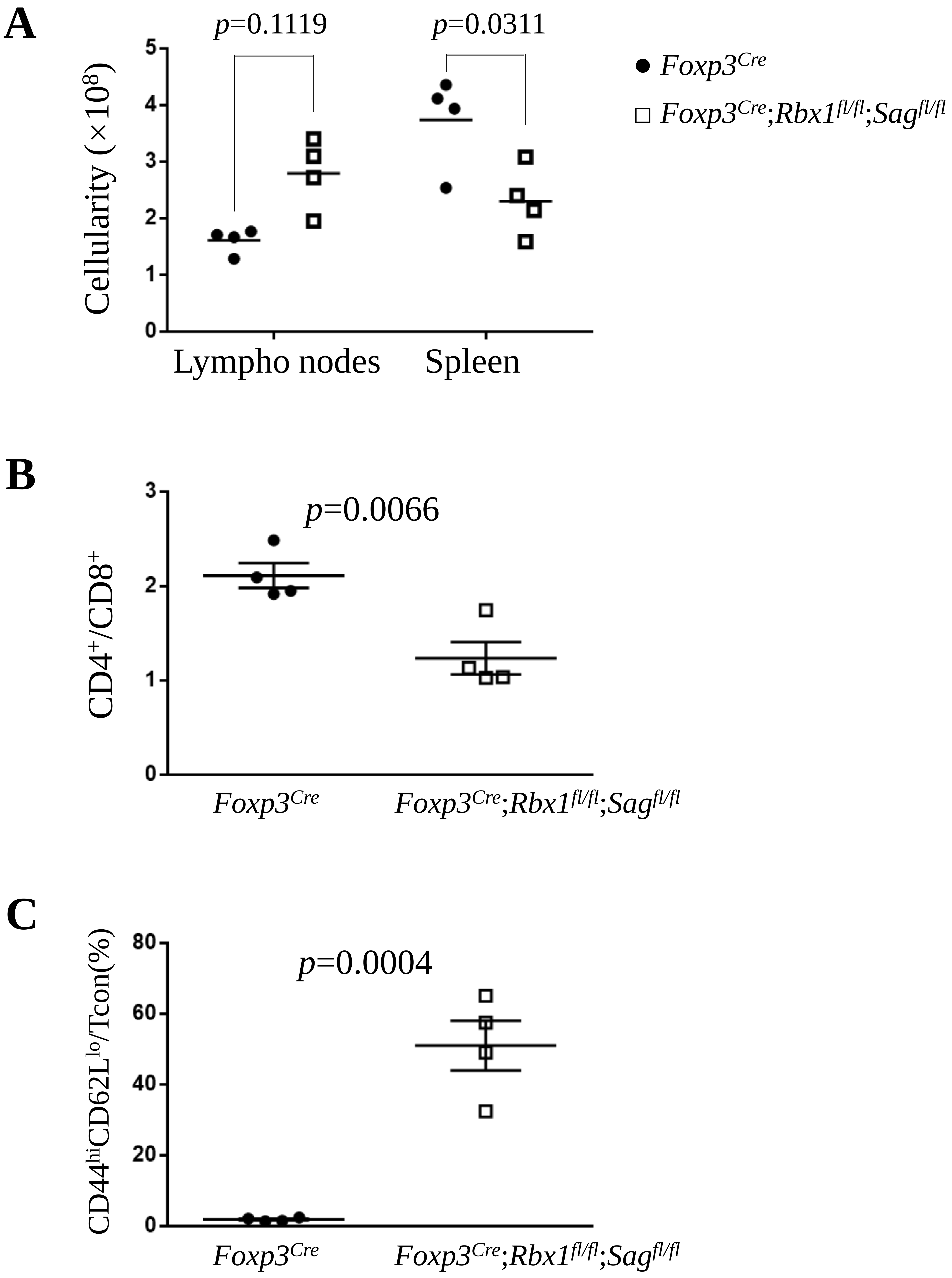

Fig. S4. Auto-immune disorders in *Foxp3*^{Cre};*Rbx1*$^{fl/fl}$;*Sag*$^{fl/fl}$ mice

(A) Total cell numbers in peripheral lymph nodes and spleen from *Foxp3*^{Cre} and *Foxp3*^{Cre};*Rbx1*$^{fl/fl}$;*Sag*$^{fl/fl}$ mice (p19-20, n=4).

(B) $CD4^+/CD8^+$ ratios in peripheral lymph nodes from *Foxp3*^{Cre} and *Foxp3*^{Cre};*Rbx1*$^{fl/fl}$;*Sag*$^{fl/fl}$ mice (p19-20, n=4).

(C) The proportion of $CD44^{hi}CD62L^{lo}$ effector/memory cells among Tcon cells in peripheral lymph nodes from *Foxp3*^{Cre} and *Foxp3*^{Cre};*Rbx1*$^{fl/fl}$;*Sag*$^{fl/fl}$ mice (p19-20, n=4).

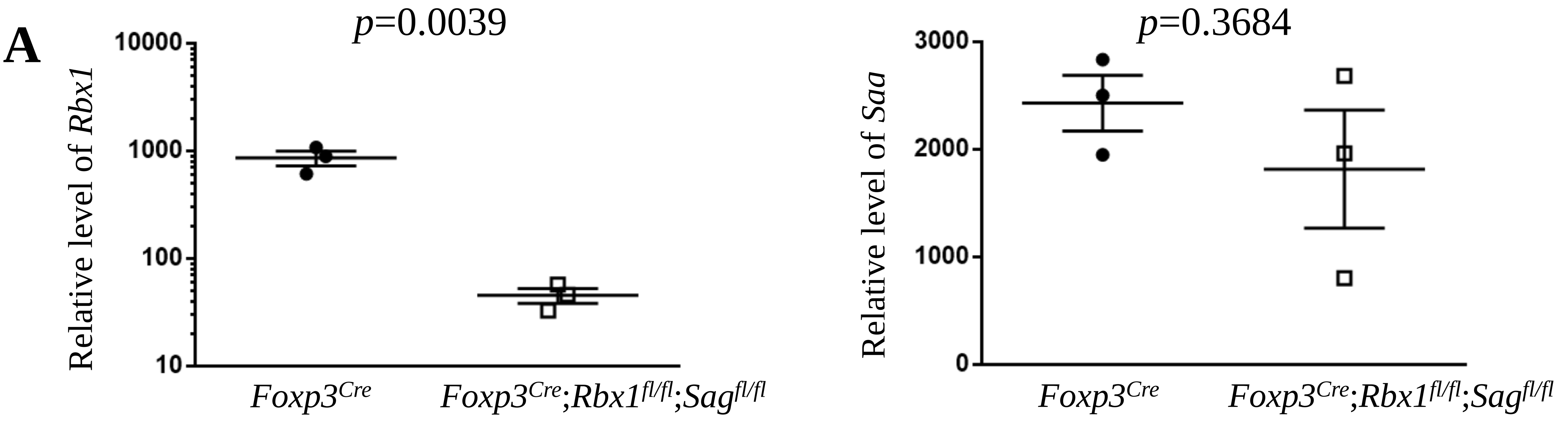

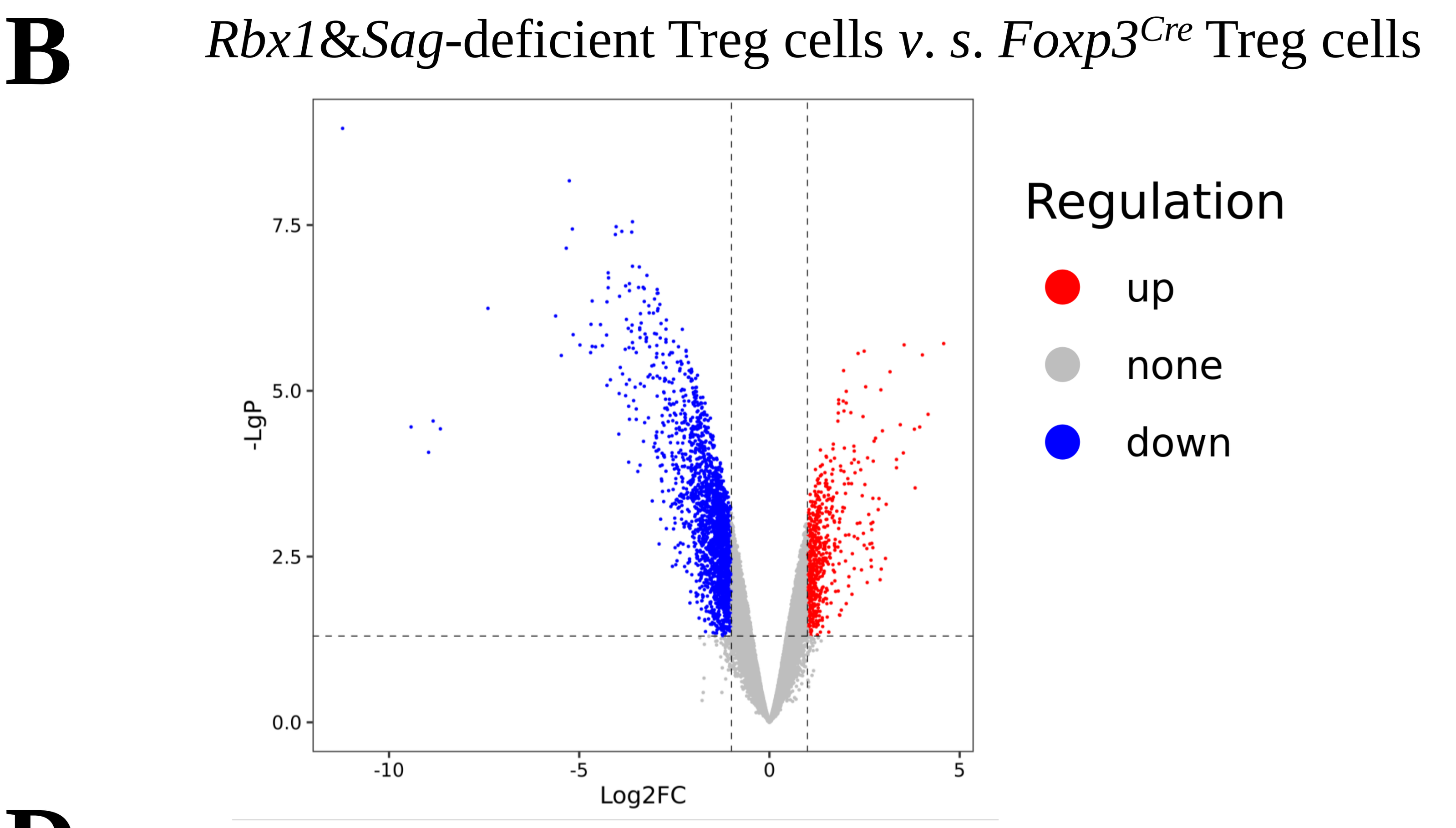

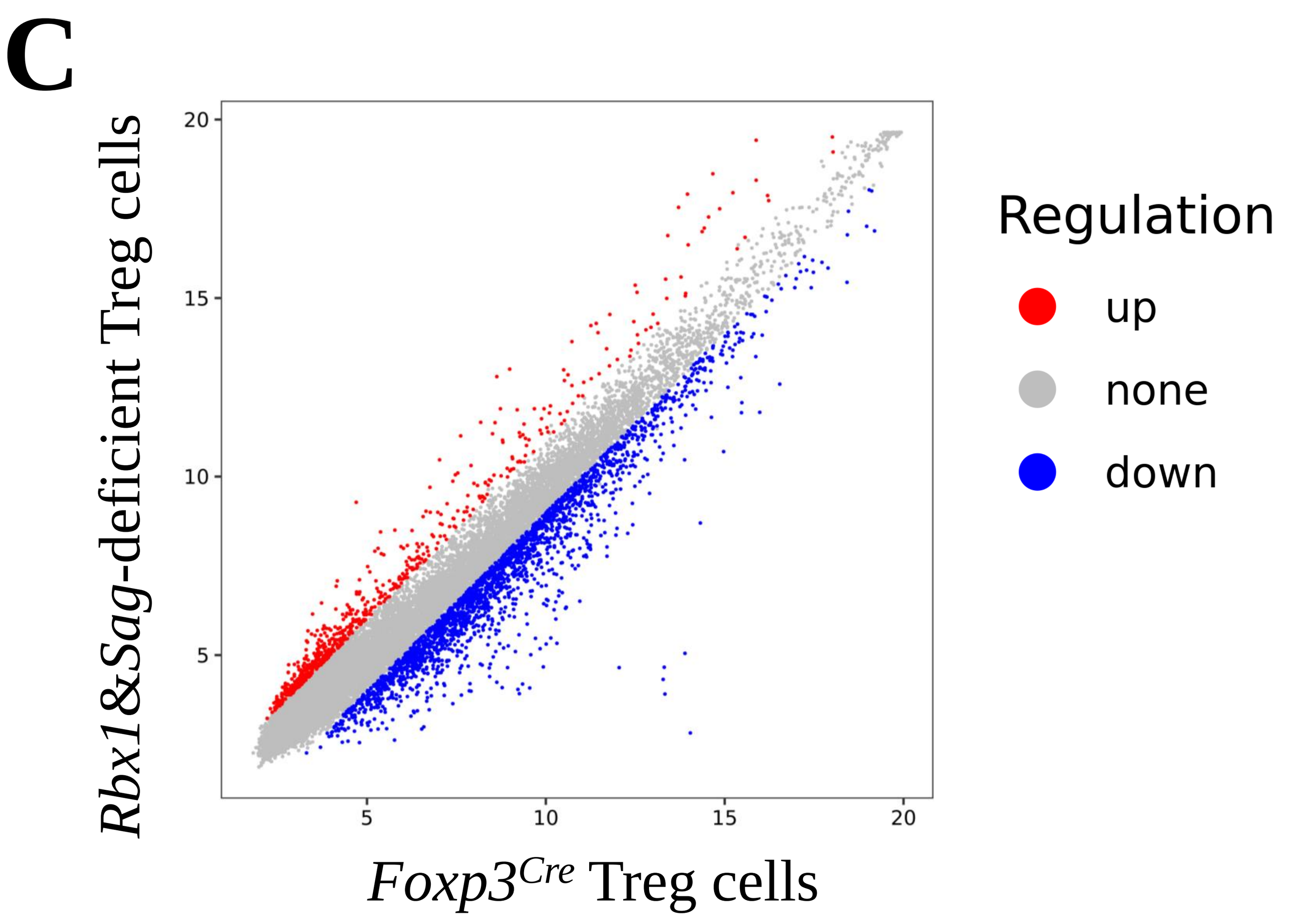

D

Gene symbol	*Foxp3*$^{Cre/wt}$ (average, log2)	*Foxp3*$^{Cre/wt}$;*Rbx1*$^{fl/fl}$;*Sag*$^{fl/fl}$ (average, log2)	Fold change
Plk2	4.70	9.28	23.918
Sik1	8.63	12.8	18.001
Nr4a2	8.99	13.01	16.223
Dusp10	13.96	17.91	15.455
Gm10715	13.71	17.54	14.221
Tnfaip3	14.67	18.48	14.026
ND6	15.88	19.42	11.632
Irs2	7.62	11.14	11.472
Ppp1r15a	7.03	10.47	10.853
Csrnp1	8.18	11.52	10.126
Gm17535	13.41	16.75	10.126
Gm10382	8.73	11.9	9.000
Ets2	5.38	8.45	8.398
Zfand2a	10.73	13.78	8.282
Nr4a1	11.26	14.23	7.835
Arrdc3	6.76	9.70	7.674
Gm11175	8.58	11.51	7.621
Gm17651	4.17	7.08	7.516
Dnajb9	11.41	14.29	7.362
Dusp5	12.5	15.36	7.260

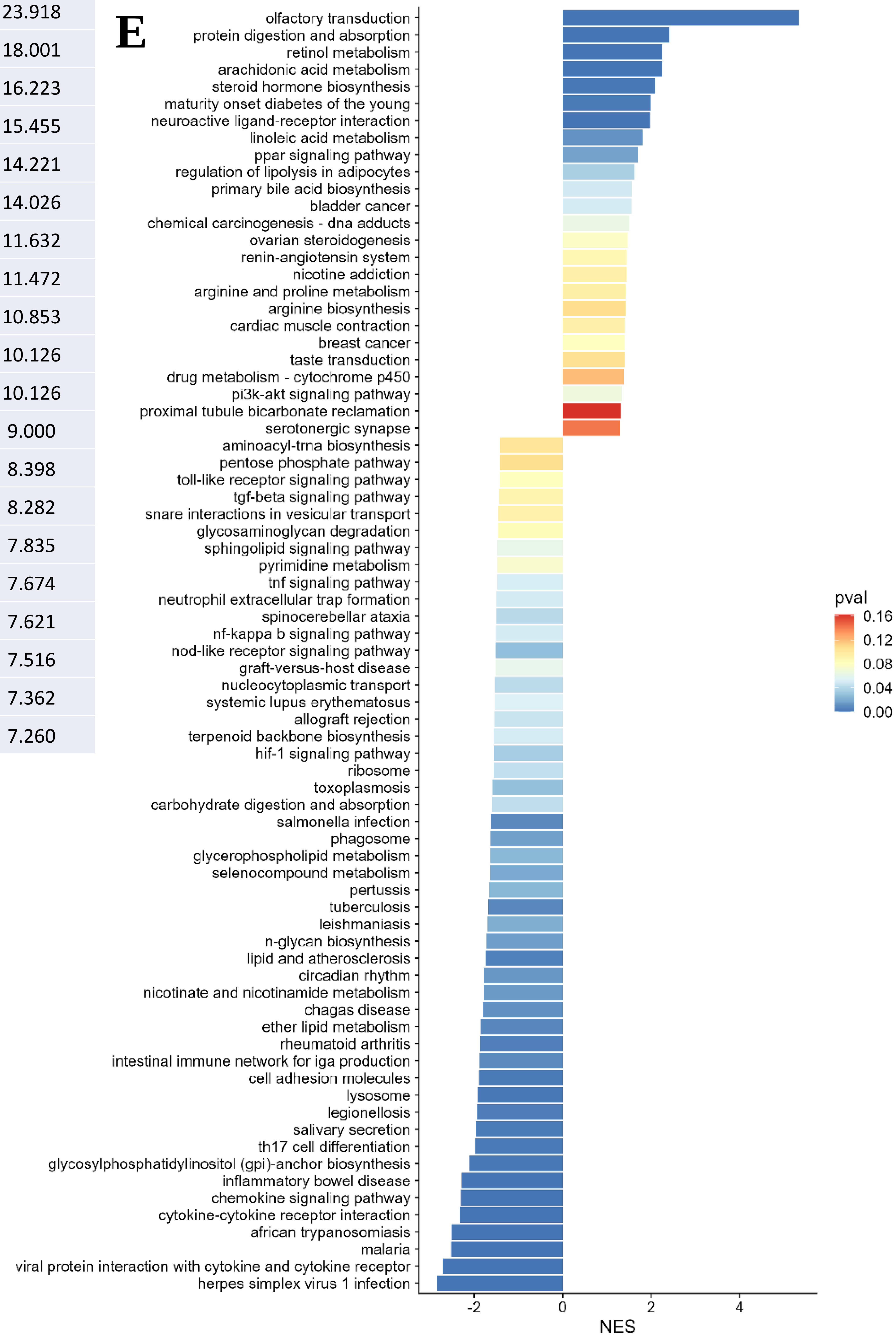

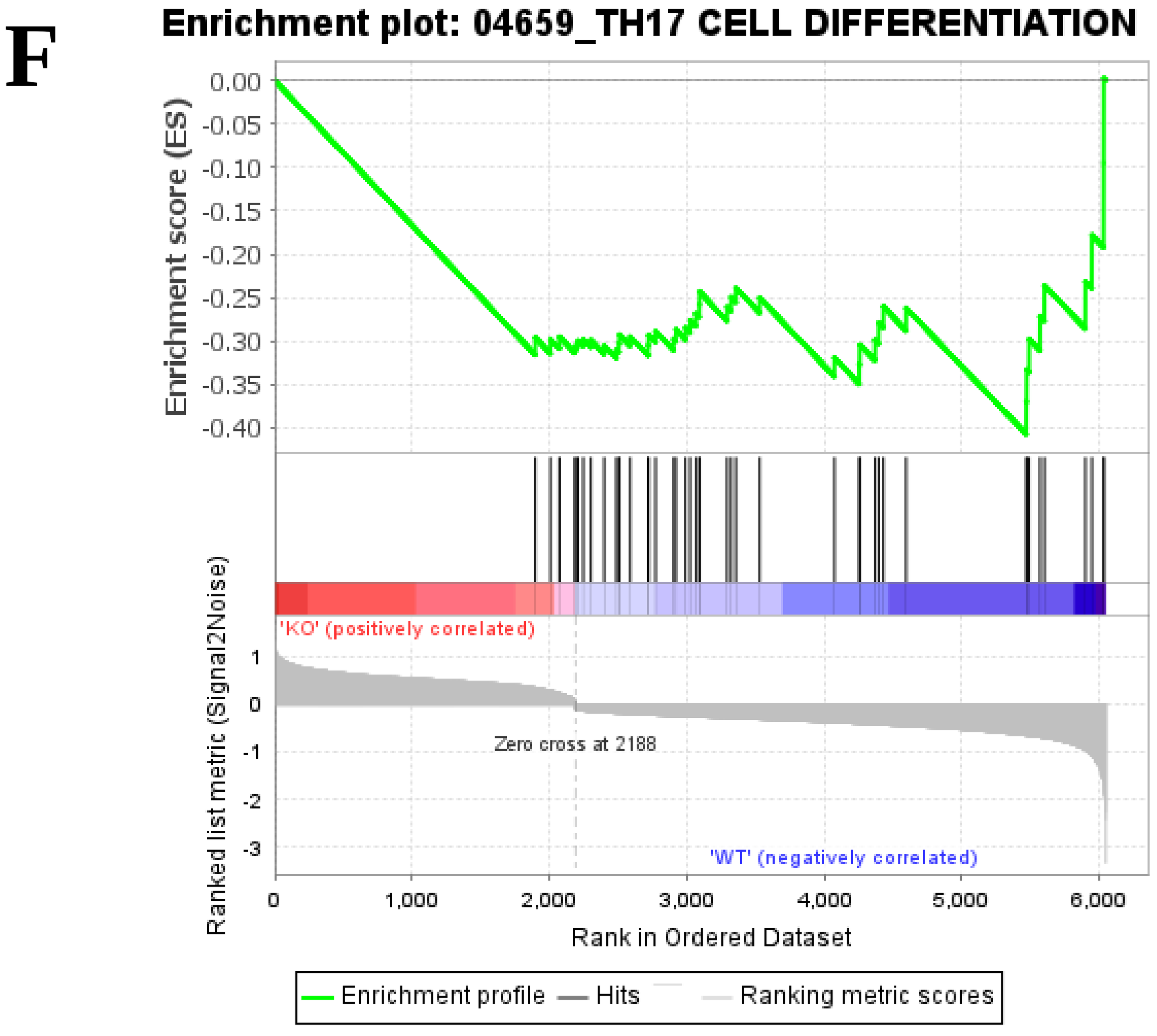

Fig. S5. Transcriptional alterations in *Rbx1*&*Sag*-deficient Treg cells

(A) Transcriptional profiling revealed dramatic decreased levels of *Rbx1* and *Sag* in *Rbx1*&*Sag*-deficient Treg cells.

(B) Volcano plot of the transcriptional alterations in *Rbx1*&*Sag*-deficient Treg cells compared to the *Foxp3*^{Cre} control Treg cells.

(C) Scatter plot of the transcriptional programs revealed from *Rbx1*&*Sag*-defificient Treg cells compared to the *Foxp3*^{Cre} control Treg cells.

(D) Top 20 up-regulated genes in *Rbx1*&*Sag*-deficient Treg cells compared to the *Foxp3*^{Cre} control Treg cells with alters more than 1.5-folds and *p*-values<0.05.

(E) GSEA pathway analysis of the transcriptional alterations in *Rbx1*&*Sag*-deficient Treg cells compared to the *Foxp3*^{Cre} control Treg cells; genes with alterations more than 1.5-folds, with *p*-values<0.05 were selected.

(F) Gene set enrichment analysis of T_H17 cell differentiation genes in $CD4^+YFP^+$ Treg cells from *Foxp3*$^{Cre/wt}$ and *Foxp3*$^{Cre/wt}$;*Rbx1*$^{fl/fl}$;*Sag*$^{fl/fl}$ mice, determined by transcriptional profiling with Fc>1.5 and $p<0.05$.

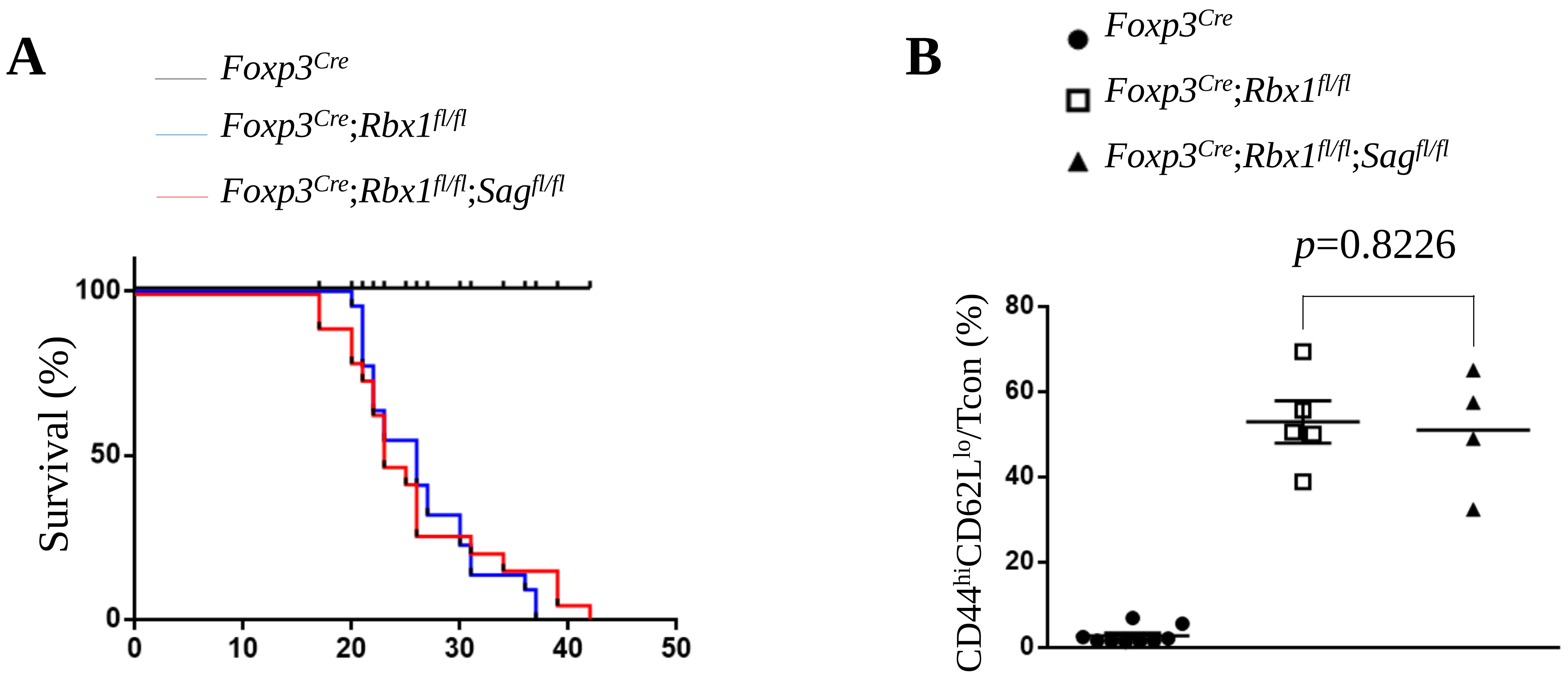

Fig. S6. Comparison of the auto-immune disorders caused by deletion of *Rbx1* or *Rbx1&Sag* in Treg cells

(A) Survival curve of *Foxp3*^{Cre}, *Foxp3*^{cre};*Rbx1*$^{fl/fl}$ and *Foxp3*^{cre};*Rbx1*$^{fl/fl}$;*Sag*$^{fl/fl}$ mice.

(B) The proportion of effector/memory cells (CD44^{hi}CD62L^{lo}) among Tcon cells in peripheral lymph nodes from *Foxp3*^{Cre}, *Foxp3*^{Cre};*Rbx1*$^{fl/fl}$ and *Foxp3*^{Cre};*Rbx1*$^{fl/fl}$;*Sag*$^{fl/fl}$ mice (p19-23).

A

Gene symbol	*Foxp3*$^{Cre/wt}$ (average, log2)	*Foxp3*$^{Cre/wt}$;*Rbx1*$^{fl/fl}$;*Sag*$^{fl/fl}$ (average, log2)	Fold change
Sik1	8.27	12.97	25.877
Csrnp1	7.30	11.70	21.035
Ppp1r15a	6.50	10.84	20.267
Plk2	4.89	9.19	19.806
Tnfaip3	14.87	18.97	17.156
Nr4a2	9.65	13.67	16.298
Dusp10	13.43	17.43	15.963
Dusp5	12.01	15.79	13.811
Zbtb10	5.48	9.11	12.367
Zfand2a	11.07	14.64	11.877
Rgs2	11.28	14.83	11.662
Hexim1	8.88	12.18	9.826
Ets2	5.52	8.79	9.657
Irs2	7.99	11.15	8.946
Six1	4.34	7.49	8.885
Stard9	8.92	12.07	8.841
Gm17535	13.71	16.84	8.795
Nr4a1	11.19	14.28	8.513
Klf6	11.86	14.87	8.093
Rgcc	5.88	8.87	7.911

B

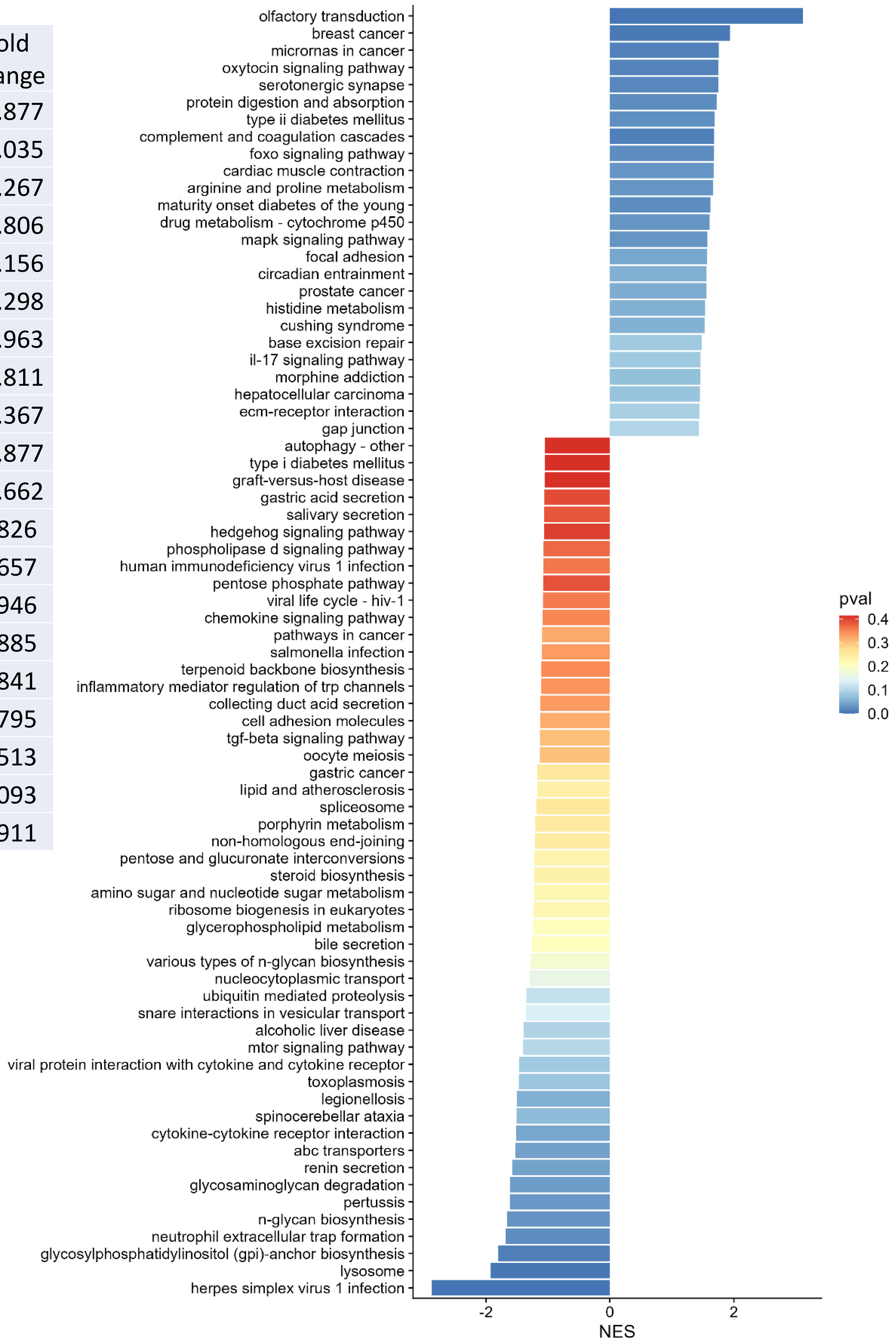

Fig. S7. Comparison of the signal alterations caused by deletion of *Rbx1* or *Rbx1&Sag* in Treg cells

(A) Top 20 up-regulated genes in *Rbx1*&*Sag*-deficient, but not *Rbx1*-deficient, Treg cells.

(B) GSEA pathway analysis of genes altered in *Rbx1*&*Sag*-deficient, but not *Rbx1*-deficient Treg cells.

A

Gene symbol	*Foxp3*$^{Cre/wt}$ (average, log2)	*Foxp3*$^{Cre/wt}$;*Rbx1*$^{fl/fl}$;*Sag*$^{fl/fl}$ (average, log2)	Fold change
ND6	13.75	19.04	39.124
Ppp1r15a	6.09	11.01	30.274
Sik1	8.07	12.86	27.665
Csrnp1	7.43	12.18	26.909
Plk2	5.18	9.77	24.084
Gm10382	7.81	12.21	21.112
Gm11175	7.11	11.45	20.252
Dusp10	13.4	17.63	18.765
Gm2174	6.82	10.93	17.268
Nr4a2	9.63	13.72	17.030
Tnfaip3	14.71	18.70	15.889
Dusp5	11.7	15.67	15.671
Zfand2a	10.71	14.63	15.137
Irs2	7.65	11.32	12.729
Gm10135	9.62	13.07	10.928
Klf2	9.92	13.32	10.556
Dusp4	6.83	10.18	10.196
Nr4a1	10.94	14.26	9.987
Maff	3.31	6.63	9.987
Rgcc	5.78	9.10	9.987

B

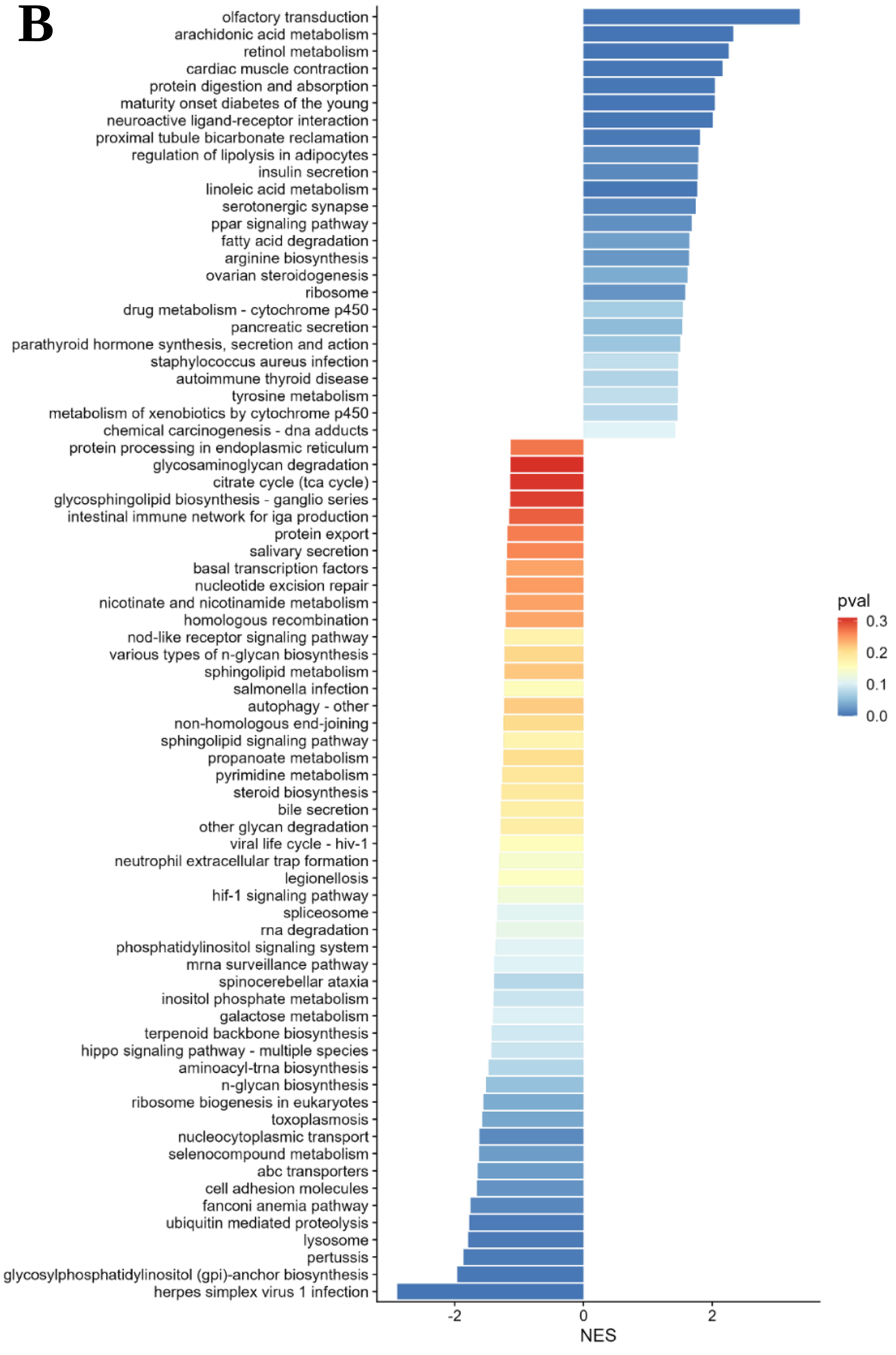

C

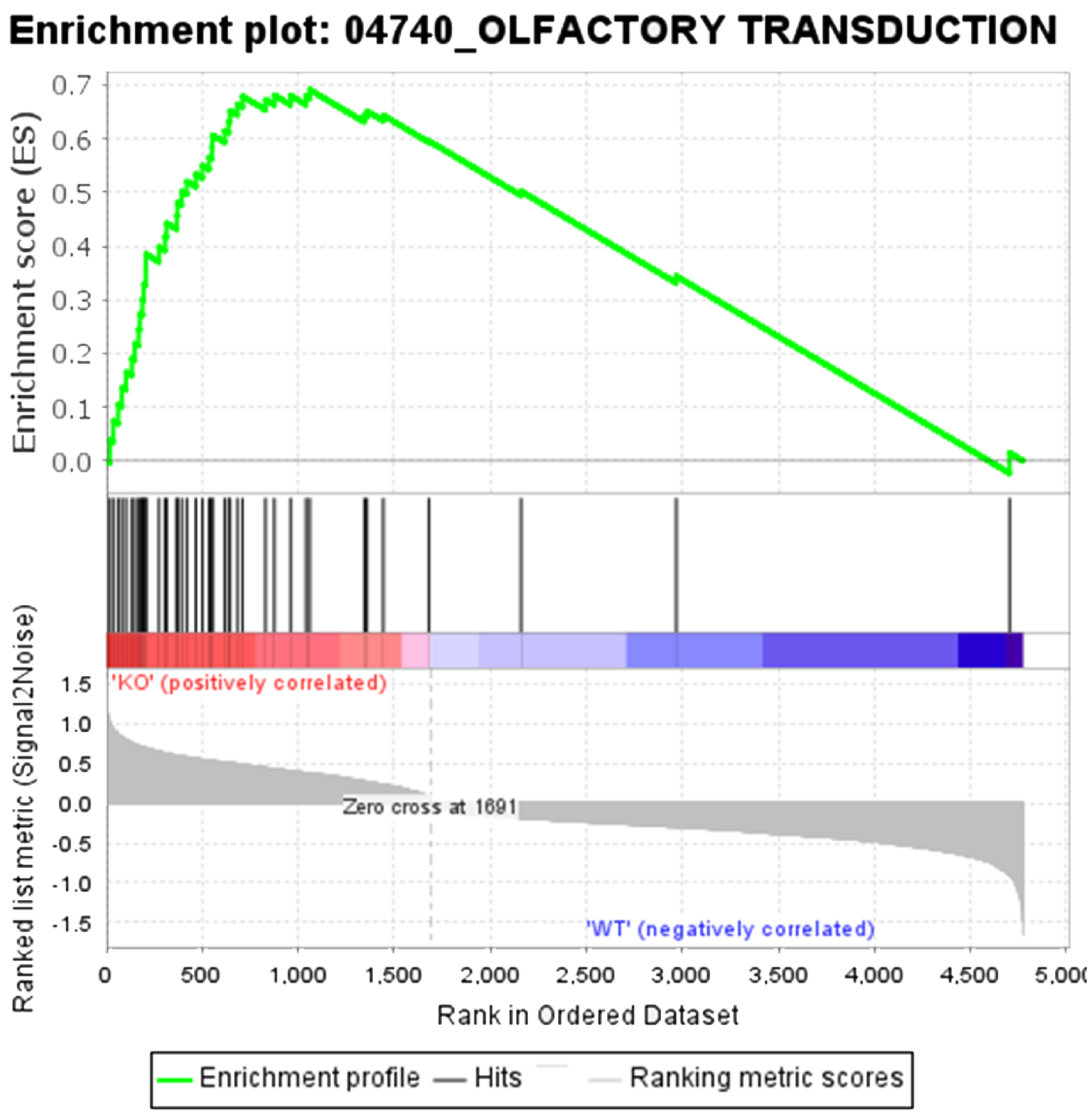

Fig. S8. GSEA pathway analysis of genes selectively altered in *Rbx1*&*Sag*-deficient, but not *Ube2m*&*Ube2f*-deficient Treg cells

(A) Top 20 up-regulated genes in *Rbx1*&*Sag*-deficient, but not *Ube2m*&*Ube2f* -deficient, Treg cells.

(B) GSEA pathway analysis of genes altered in *Rbx1*&*Sag*-deficient, but not *Ube2m*&*Ube2f*-deficient Treg cells.

(C) Gene set enrichment analysis of olfactory transduction genes in genes altered in *Rbx1*&*Sag*-deficient, but not *Ube2m*&*Ube2f*-deficient Treg cells.

A

Gene symbol	*Foxp3*$^{Cre/wt}$ (average, log2)	*Foxp3*$^{Cre/wt}$;*Ube2m*$^{fl/fl}$;*Ube2f*$^{fl/fl}$ (average, log2)	Fold change
Rgs16	10.34	5.96	-20.821
Ube2m	12.43	8.43	-16.000
Lgals1	14.16	10.66	-11.314
1500004A13Rik	8.67	5.66	-8.056
Rln3	8.84	5.85	-7.945
Rilpl2	8.99	6.35	-6.233
Il18rap	7.61	5.14	-5.540
Pglyrp1	9.17	6.77	-5.278
Ctla2a	7.68	5.28	-5.278
Ptprs	10.05	7.69	-5.134
Dmbt1	6.50	4.14	-5.134
Nfkbia	15.07	12.8	-4.823
Ube2f	12.19	9.93	-4.790
Itm2c	11.94	9.78	-4.469
Tesc	6.37	4.37	-4.000
Ccrl2	7.46	5.48	-3.945
Timp2	10.03	8.08	-3.864
Crip1	11.52	9.59	-3.811
Fbxw8	7.55	5.67	-3.681
Odc1	9.54	7.67	-3.655

B

Gene symbol	*Foxp3*$^{Cre/wt}$ (average, log2)	*Foxp3*$^{Cre/wt}$;*Ube2m*$^{fl/fl}$;*Ube2f*$^{fl/fl}$ (average, log2)	Fold change
Pde3b	5.43	9.22	13.833
Chil5	5.87	8.75	7.362
Parp8	10.44	13.2	6.774
Gm3123	3.76	6.26	5.657
Gm8127	4.58	7.03	5.464
Gm10721	17.64	19.73	4.257
Txnrd1	9.40	11.41	4.028
Elovl6	6.72	8.63	3.758
Trib2	12.8	14.68	3.681
Gm2310	11.84	13.67	3.555
Myb	9.82	11.59	3.411
Ms4a4c	14.28	16.02	3.340
Cers6	8.97	10.71	3.340
Gm8068	4.56	6.24	3.204
Gm8005	4.38	6.00	3.074
Olfr771	3.90	5.52	3.074
Txk	11.58	13.12	2.908
Gm826	7.81	9.34	2.888
Cln3	9.47	10.99	2.868
Gm10576	9.24	10.75	2.848

C

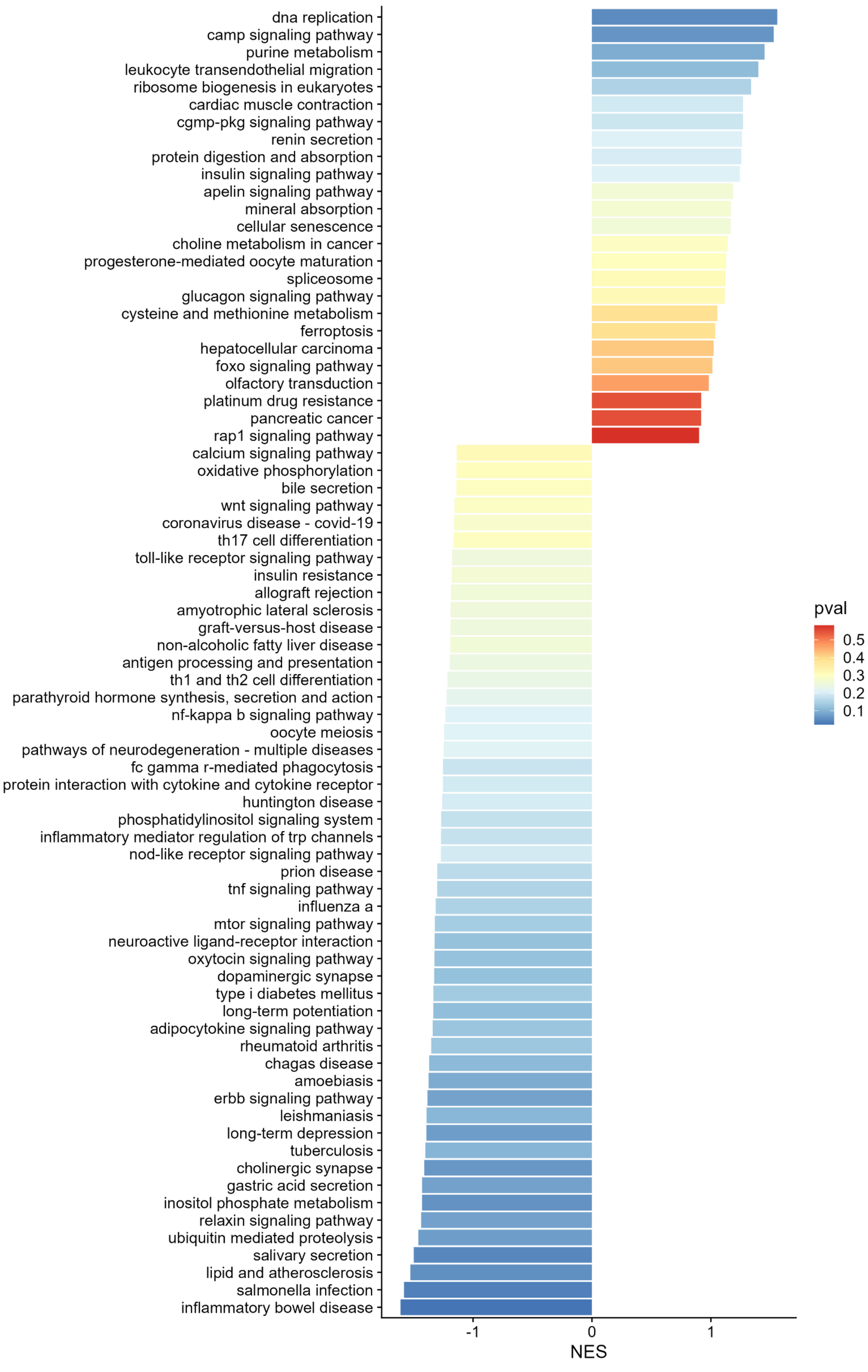

D

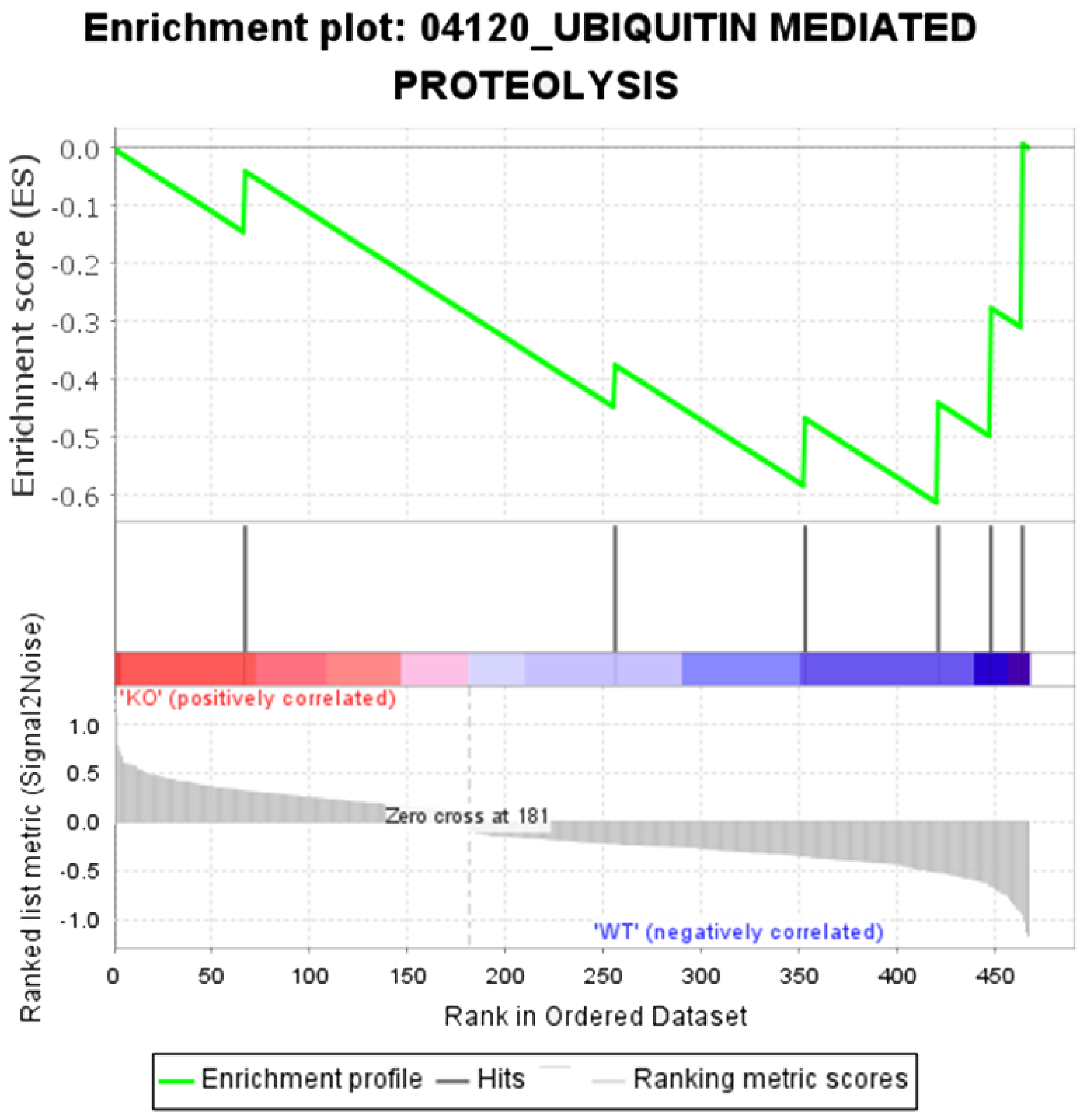

E

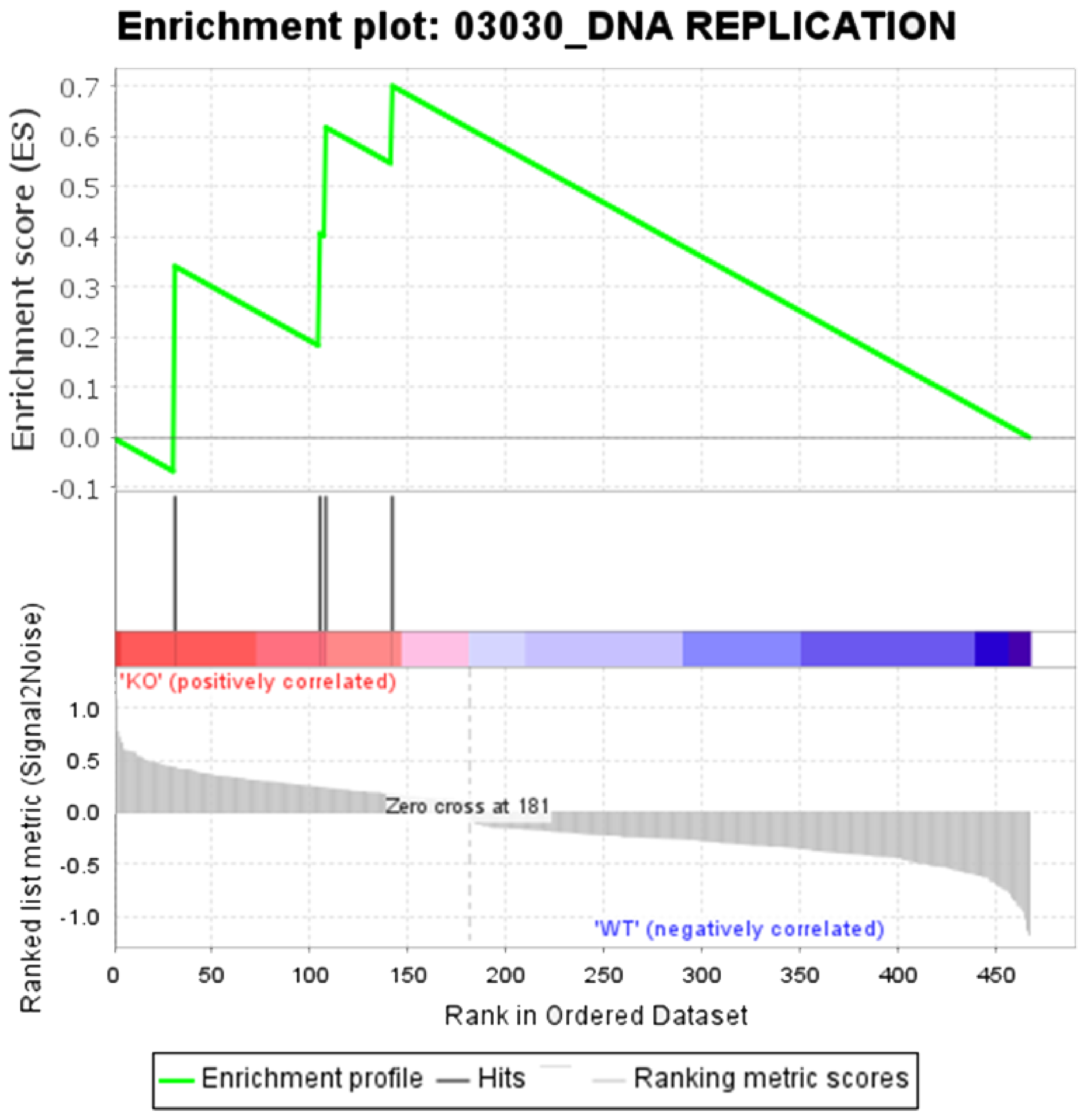

Fig. S9. GSEA pathway analysis of genes selectively altered in *Ube2m*&*Ube2f*-deficient, but not *Rbx1*&*Sag*-deficient Treg cells

(A) Top 20 down-regulated genes in *Ube2m*&*Ube2f*-deficient, but not *Rbx1*&*Sag*-deficient, Treg cells.

(B) Top 20 up-regulated genes in *Ube2m*&*Ube2f*-deficient, but not *Rbx1*&*Sag*-deficient, Treg cells.

(C) GSEA pathway analysis of genes altered in *Ube2m*&*Ube2f*-deficient, but not *Rbx1*&*Sag*-deficient Treg cells.

(D) Gene set enrichment analysis of ubiquitin mediated proteolysis genes in genes altered in *Ube2m*&*Ube2f*-deficient, but not *Rbx1*&*Sag*-deficient Treg cells.

(E) Gene set enrichment analysis of DNA replication genes in genes altered in *Ube2m*&*Ube2f*-deficient, but not *Rbx1*&*Sag*-deficient Treg cells.
